# PCV2 Regulates Cellular Inflammatory Responses through Dysregulating Cellular miRNA-mRNA Networks

**DOI:** 10.3390/v11111055

**Published:** 2019-11-13

**Authors:** Chang Li, Yumei Sun, Jing Li, Changsheng Jiang, Wei Zeng, Hao Zhang, Shengxian Fan, Qigai He

**Affiliations:** 1 State Key Laboratory of Agricultural Microbiology, Huazhong Agricultural University, Wuhan 430000, China; lichang1113@webmail.hzau.edu.cn (C.L.); Lijing789@webmail.hzau.edu.cn (J.L.); changshengjiang@webmail.hzau.edu.cn (C.J.); aiyouwei@webmail.hzau.edu.cn (W.Z.); zhanghao@webmail.hzau.edu.cn (H.Z.); 2 The Cooperative Innovation Center for Sustainable Pig Production, Huazhong Agricultural University, Wuhan 430000, China; fanshengxian@mail.hzau.edu.cn; 3 College of Veterinary Medicine, Huazhong Agricultural University, Wuhan 430000, China; sym@webmail.hzau.edu.cn

**Keywords:** porcine circovirus type 2, porcine kidney 15 cell line, microRNAs, interaction network, inflammation

## Abstract

Porcine circovirus type 2 (PCV2) is closely linked to postweaning multisystemic wasting syndrome (PMWS) and other PCV-associated diseases (PCVADs), which influence the global pig industry. MicroRNAs (miRNAs) are evolutionarily conserved classes of endogenous small non-coding RNA that regulate almost every cellular process. According to our previous transcription study, PCV2 infection causes up-regulation of genes related to inflammation. To reveal the function of miRNAs in PCV2 infection and PCV2-encoded miRNAs, next generation sequencing and data analysis was performed to explore miRNA expression in PCV2-infected cells and non-infected cells. Data analysis found some small RNAs matched the PCV2 genome but PCV2 does not express miRNAs in an in vitro infection (PK-15 cells). More than 297 known and 427 novel miRNAs were identified, of which 44 miRNAs were differently expressed (DE). The pathways of inflammation mediated by chemokine and cytokine signaling pathway (P00031), were more perturbed in PCV2-infected cells than in mock controls. DE miRNAs and DE mRNA interaction network clearly revealed that PCV2 regulates the cellular inflammatory response through dysregulating the cellular miRNA-mRNA network. MiRNA overexpression and down-expression results demonstrated that miRNA dysregulation could affect PCV2 infection-induced cellular inflammatory responses. Our study revealed that host miRNA can be dysregulated by PCV2 infection and play an important role in PCV2-modulated inflammation.

## 1. Introduction

PCV belongs to the genus *Circovirus* and family *Circoviridae*, and currently, three porcine circovirus genotypes have been reported [1,2,3]. PCV2 has been identified as the main cause of PCVADs, such as postweaning multisystemic wasting syndrome, porcine dermatitis and nephropathy syndrome, and porcine respiratory disease complex [4,5]. Our previous studies have reported a correlation between a deregulated immune-inflammatory response and cytokine dysfunction in infected pigs and cells [6]. Nevertheless, understanding of the mechanisms underlying the deregulation of immune responses remains limited.

MiRNAs can regulate translation by binding to complementary regulatory RNA sequences, thereby causing mRNA degradation to regulate biological processes such as differentiation [7], proliferation [8], growth [9], metabolism [10,11], and apoptosis [12]. The study of miRNA-mediated host-pathogen interactions has emerged in the last decade due to the increasing knowledge regarding the role that miRNAs play in antiviral defense. There is increasing evidence indicating that viruses modulate cellular miRNA expression profiles upon host infection. The altered expression of cellular miRNAs may facilitate and/or restrict viral replication by deregulating their target genes after viral infections [13]. No miRNAs encoded by PCV2 genomic DNA were detected in tonsil and mediastinal lymph node tissues from PCV2-infected pigs [14], hence, PCV2 may completely invade and colonize other life processes by regulating host miRNAs. The miRNA expression pattern in PCV2 of sub-clinically infected pigs, non-infected pigs, and PK-15 cells expressing each of the three PCV2-encoded open reading frames (ORFs) have been reported [15,16,17]. However, there is limited information on miRNA expression profiling in PCV2-infected cells. The correlation between miRNA and inflammatory response associated genes remain unknown. Whether PCV2 encodes miRNAs needs to be evaluated using deeper sequencing.

**Figure 1:**
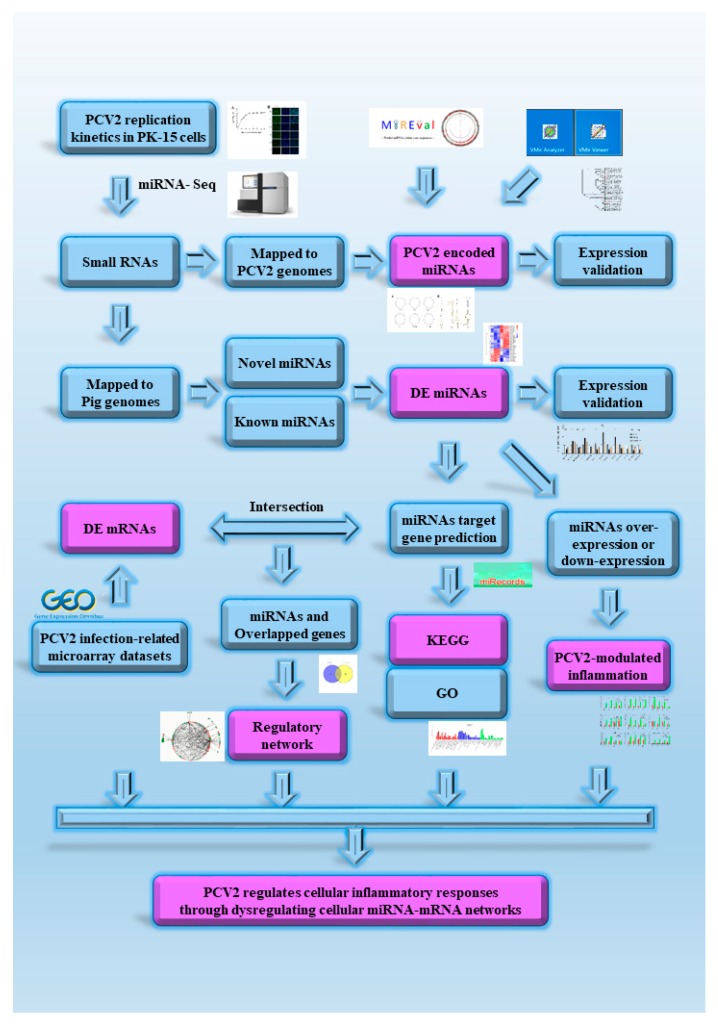
Flow chart of the present study. PCV2: Porcine circovirus type 2; GEO: Gene Expression Omnibus; DE: Differently expressed; GO: Gene Oncology; KEGG: Kyoto Encyclopedia of Genes and Genomes.

The flow chart recapitulating the present work is shown in Figure 1. In this study, miRNA expression profiles in PCV2-infected and non-infected cells were determined by RNA sequencing; the deeper sequencing results were also used to reassess whether PCV2 could encode miRNAs. Some small RNAs matched with the PCV2 genome but the qPCR results showed that PCV2 does not express miRNAs in an in vitro infection (PK-15 cells). The interaction network analysis was performed by analyzing our miRNA sequencing results and the published PCV2 transcriptome. The network analysis, pathway analysis, and miRNA expression experiment results clearly revealed that PCV2 regulates cellular inflammatory responses through dysregulating the cellular miRNA-mRNA network. In summary, our study provides a valuable basis for further investigation of the functions of miRNA in PCV2 pathogenesis.

## 2. Materials and Methods

### 2.1. Cells and Virus

PK-15 cell line were cultured in Dulbecco’s modified Eagle medium (DMEM, Gibco, Thermo Fisher Scientific, Waltham, MA, USA) supplemented with 10% heat-inactivated fetal bovine serum (FBS, Natocor, Cordoba, ARG) at 37 °C in a humidified incubator containing 5% CO2. The PCV2 strain (GenBank accession no. FJ598044.1), which belongs to the PCV2b gene subtype was isolated from a pig farm in the Hubei province of China.

### 2.2. Cell Culture and Treatment

For virus infection and titration, PK-15 cells grown to approximately 70%–90% confluence were infected with PCV2 at a multiplicity of infection (MOI) of 0.1, 1.0, 2.0, 5.0, and 10.0. After 1 h of adsorption, infected cells were maintained in DMEM supplemented with 2% FBS for 24 h. Viral infection was confirmed with immunofluorescence (IFA). The IFA was performed using cold absolute ethanol as the fixative and pig anti-PCV2 polyclonal antiserum (VMRD, USA) as the primary antibody.

For transfection, PK-15 cells grown to approximately 30%–50% confluence were transfected with the miRNA mimics (50 nmol/well), miRNA mimics control (50 nmol/well), inhibitors (50 nmol/well), and inhibitors control (50 nmol/well) purchased from GenePharma (Shang, CHN) with Lipofectamine 2000 (Life Technologies, CA, USA). The sequences of the mimics, inhibitors, or scrambled oligonucleotides are listed in Supplementary Table S1.

### 2.3. RNA Isolation, Sequencing, and Data Analysis

Total RNA from PCV2-infected (*n* = 3) (MOI = 10) and mock-infected (*n* = 3) (MOI = 0) cells was isolated by using Trizol (Invitrogen, Carlsbad, CA, USA) at 24 h past infection and its purity, concentration, and integrity were evaluated. All RNA preparations had a ratio of absorbance (260/280 nm) > 1.8 and RNA integrity numbers (RINs) > 7.

Each small RNA library used 3 μg total RNA as input material. The sequencing libraries were generated using NEBNext^®^ Multiplex Small RNA Library Prep Set for Illumina^®^ (New England Biolabs (Beijing) LTD, Beijing, CHN) and were sequenced on an Illumina Hiseq 2500 platform to generate 50 bp single-end reads. The raw reads were processed through custom perl and python scripts to obtain clean read lengths ranging from 18–35 nt. The small RNA tags were mapped to the sus scrofa genome sequence by Bowtie [18] without mismatch to analyze their expression and distribution on the reference.

The small RNA tags were mapped to the PCV2WH genome sequence by Bowtie without mismatch to find small RNAs expression and distribution in the PCV2 genome. miREval 2.0 [19] and VMir [20] were also used to explore PCV2 encoded miRNAs.

Mapped small RNA tags were used to find known miRNA using miRBase 20.0 as a reference. Mirdeep2 [21] and srna-tools-cli were used to obtain the potential miRNA. MiRNA expression levels were estimated by TPM (transcript per million) [22]: Normalization formula: Normalized expression = mapped readcount/Total reads*1,000,000. Differential expression analysis (PCV2-infected and mock-infected) was performed using the DESeq R package (1.8.3). The *p*-values were adjusted using the Benjamini and Hochberg method. A corrected P-value of 0.05 was set as the threshold for significantly differential expression by default.

### 2.4. Validation of DE miRNAs/mRNA with the RT-qPCR Method

For the miRNA detection, total RNA was reverse-transcripted into single strand cDNA by specific stem-loop primers using PrimeScript™ RT reagent Kit RR037A (TaKaRa, Shiga-ken, Japan). HieffTM qPCR SYBR^®^ Green Master Mix (Low Rox Plus) (YEASEN, ShangHai, CHN) was used to perform real-time PCR. The U6 small nuclear RNA (snRNA) was used as an endogenous control.

For the cytokine mRNA detection, total RNA was reverse-transcripted into single strand cDNA using PrimeScript™ RT reagent Kit RR036A (TaKaRa, Shiga-ken, Japan). The real-time PCR was conducted as described above. The GAPDH gene was used as the internal control.

MiRNA or mRNA expression were determined using the 2^−ΔΔCT^. *p*-values < 0.05 were considered statistically significant. Oligonucleotides used as primers for miRNA and mRNA qRT-PCR analysis are displayed in Supplementary Table S2.

### 2.5. MiRNAs Target Prediction and Functional Enrichment Analysis

The DE miRNA target genes prediction was performed using miRecords [23], which involves 11 predicted algorithms (Targetscan, RNAhybrid, RNA22, PITA, Pictar, NBmiRTar, mitarget, MirTarget2, miRanda, Microinspector, and DIANA-microT). Target genes forecasted by at least three algorithms were selected for further analysis. Gene ontology (GO) and the Kyoto Encyclopedia of Gene and Genome (KEGG) pathway analyses of the targets were conducted by PANTHER 14.0 [24].

### 2.6. Construction of the miRNA-mRNA Network

Microarray datasets providing mRNA expression profile in PK-15 cells infected with PCV2 were achieved from the Gene Expression Omnibus (Series GSE71945). The dataset (GSE71945) includes microarray results from 60 samples, including the mRNA expression profile results of PCV2-infected PK-15 cells and non-infected PK-15 cells. The DE mRNAs were obtained by analyzing with GEO2R. The overlapping genes between the predicted miRNA target genes and the DE mRNAs were obtained for the miRNA-mRNA network construction. Cytoscape 3.6.1 software [25] was used to visualize the regulatory network.

## 3. Results

### 3.1. PCV2 Replication Kinetics in PK-15 Cells

**Figure 2:**
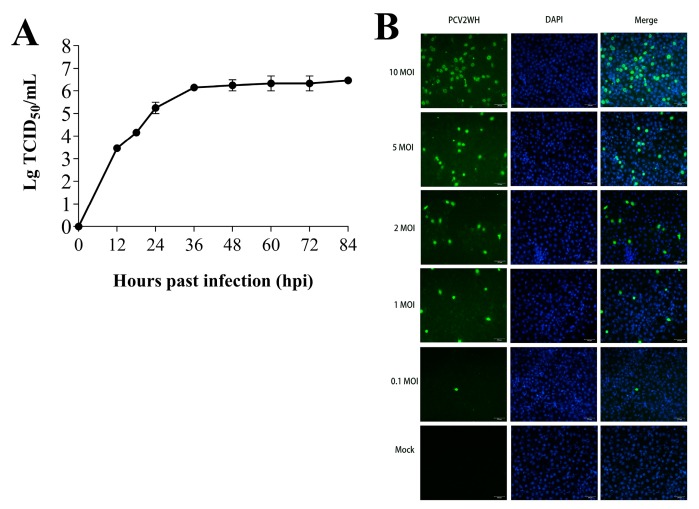
PCV2 replication kinetics in PK-15 cells. (**A**) PCV2 titers in PK-15 cells infected with PCV2 at 12, 24, 36, 48, 60, 72, 84 hpi. (**B**) The number of PCV2-positive cells in PK-15 cells after PCV2 infection at multiplicity of infection (MOI) of 0.1, 1.0, 2.0, 5.0 10.0 at 24 hpi. Green: PCV2-positive PK-15 cells; blue: DAPI (4′,6-diamidino-2-phenylindole) (/Beyotime Biotechnology, Shanghai, CHN)-stained PK-15 cell nucleus.

The replication kinetics of PCV2 in PK-15 cells were confirmed with IFA. Virus titers at different stages of infection are shown in Figure 2A. The PCV2 titers showed significant time-dependent increases and PCV2 grows fastest in the first 24 h after infection. We also evaluated the correlation between multiplicity of infection (MOI) and the number of PCV2-positive cells. There was an evident dose-dependent rise (Figure 2B). No excessive CPE was found, but with high virus levels the optimal time for miRNA expression analysis in host cells is during the early stage of virus infection, because it may trigger global and strong antiviral immune responses [26,27]. Here, PCV2-infected and mock-infected PK-15 cells were harvested at 24 hpi for further miRNA sequencing analysis to guarantee a higher proportion of infected cells and cell viability.

### 3.2. Small RNA Sequencing Analysis

To identify miRNA expression in PK-15 cells, small RNA libraries pooled from mock-infected and PCV2-infected cells were constructed. Sequencing of each library yielded 10,960,073 to 14,670,375 raw reads (Supplementary Table S3). After excluding low-quality reads and adaptor sequences, the remaining sequences of 18–35 nt in length were selected as clean reads. The percentage of clean reads was approximately 97.55%. The length distribution of the clean reads was similar in all the libraries, with the majority being 22 nt. The clean reads were mainly distributed in the sense strand of the pig genome (Supplementary Table S4) and were mainly distributed on chromosomes 1, 2, 3, 6, 8, 9, 13, 14, 15, and X. In our libraries, an average of 54.22% of the reads were miRNAs, and an average of 39.11% remained unclassified (Supplementary Table S5).

A total of 297 conserved mature miRNAs that belonged to 157 miRNA families (Supplementary Table S6) and 427 novel mature miRNAs (Supplementary Table S7) were identified. The first base of the 18~30 nt miRNAs were mainly U, and the base of the 22 nt miRNAs were mainly U, followed by A, G, and C.

**Figure 3:**
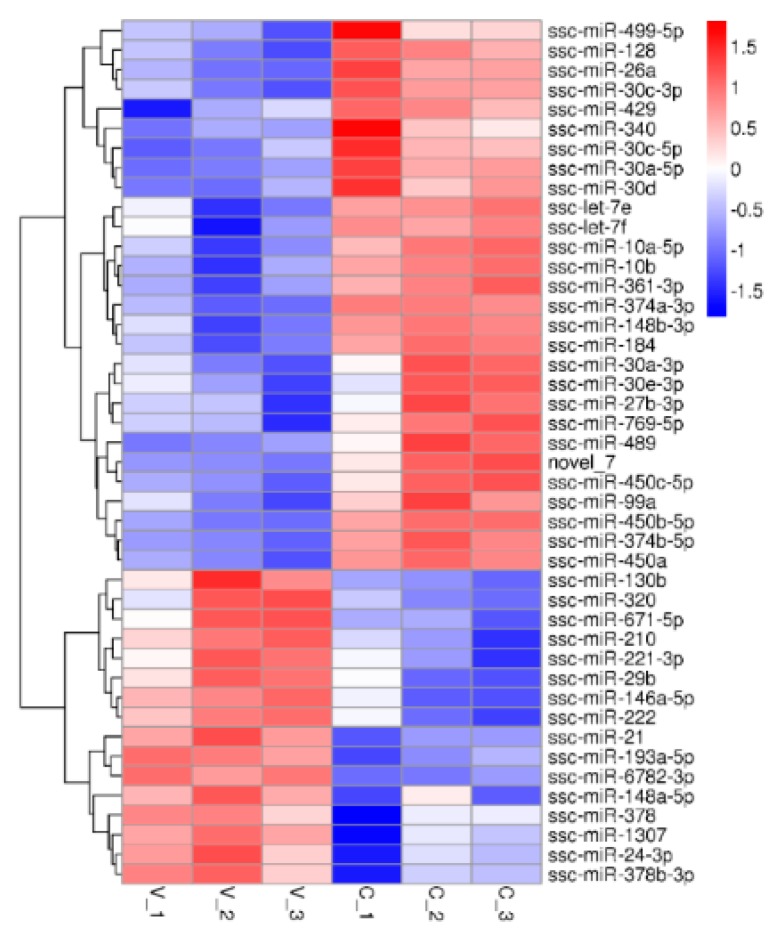
Cluster analysis of DE miRNA between the PCV2-infected and mock-infected PK-15 cells. There were 44 miRNAs (16 up-regulated and 28 down-regulated) in the PCV2-infected PK-15 cells (V_1, V_2, V_3) that exhibited differential expression (C_1, C_2, C_3).

**Figure 4:**
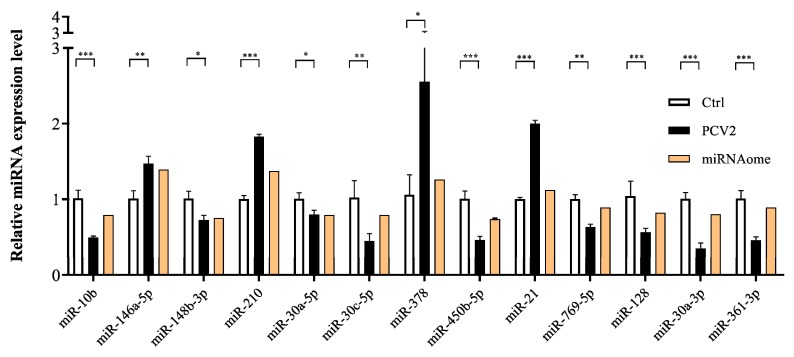
Validation of DE miRNA expression by qRT-PCR. The relative expression level of thirteen miRNA in PCV2-infected PK-15 cells were calculated using the 2^−ΔΔCT^ method and represented as the n-fold change compared to the mock-infected sample. The U6 snRNA as an endogenous control. The data were expressed as mean ± standard error of measurement (SEM). Statistical significance was determined by Student’s *t*-test, with *p*-values of < 0.05 deemed to be statistically significant (*, *p* < 0.05; **, *p* < 0.01; ***, *p* < 0.001).

MiRNA read counts were analyzed to identify DE miRNAs between PCV2-infected and mock-infected cells. A total of 44 DE miRNAs (16 up-regulated and 28 down-regulated) were identified (Figure 3) (Supplementary Table S8). To validate the differential expression of some of the DE miRNAs identified by RNA sequencing, 13 selected DE miRNAs (miR-10b, miR-146a-5, miR-148b-3p, miR-210, miR-30a-5p, miR-30c-5p, miR-378, miR-450b-5p, miR-21, miR-769-5p, miR-128, miR-30a-3p, miR-361-3p) were selected for qRT-PCR analysis. Collectively, the qRT-PCR results were consistent with the sequencing results (Figure 4).

### 3.3. PCV2 Encoded miRNA Capability Evaluation

**Figure 5:**
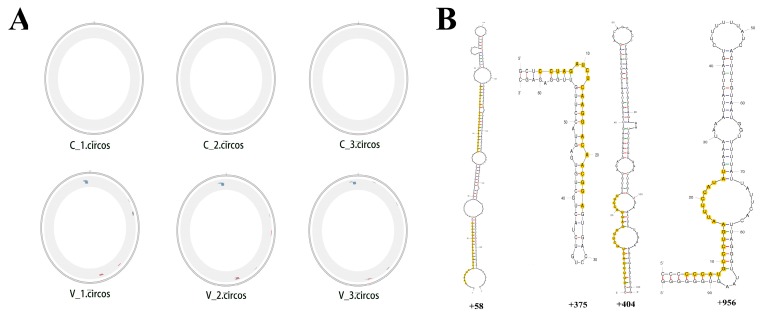
PCV2 encoded miRNAs analysis. (**A**) The distribution of mapped small RNAs in the PCV2WH genome sequences. The mapped small RNAs were mainly located in position of +799 and –1733 in the PCV2 genome sequences. (**B**) Predicted hairpin structures of potential pre-miRNAs. Yellow: the predicted putative miRNA locations in pre-miRNAs; red hyphen: Hydrogen bond between G and C.

To explore the PCV2 encoded miRNAs, the small RNA tags obtained in sequencing were mapped to the PCV2WH genome sequence. As shown in Figure 5A, some small RNAs matched with the PCV2 genome. The mapped small RNAs were mainly located in the start position of +799 and _1733, among which +1733 was at the origin of replication. The details of the mapped small RNAs are described in Supplementary Table S9. miREval 2.0 and VMir software were also used to identify potential miRNA precursors. A total of 24 miRNA-like hairpins were predicted by using miREval and its sequences and structure diagrams are shown in Supplementary Table S10. A total of 61 PCV2 whole genomes from different genotypes (PCV2a, PCV2b, PCV2c, PCV2d, PCV2e,) were used to predict PCV2-produced pre-miRNAs by using VMir. Pre-miRNAs were predicted to be mainly distributed at a start position of +58, +375, +404, and +956 in the PCV2 genome. The detailed information of each pre-miRNA is listed in Supplementary Table S11. The acquired pre-miRNA sequences were analyzed to identify mature miRNAs by using the database MatureBayes [28] and MaturePred [29]. The mfold [30] was used to predict consensus secondary structures of the pre-miRNAs. The predicted mature miRNA locations in pre-miRNAs are shown in Figure 5B. Stem-loop qPCR was performed to detect its expression in PCV2-infected cells and non-infected cells, however, expression was not detected, or expression levels were not different between the infected cells and non-infected cells. Data analysis did not find PCV2-encoded miRNAs in an in vitro infection (PK-15 cells).

### 3.4. DE miRNA Target Genes Prediction and Functional Analysis

**Figure 6:**
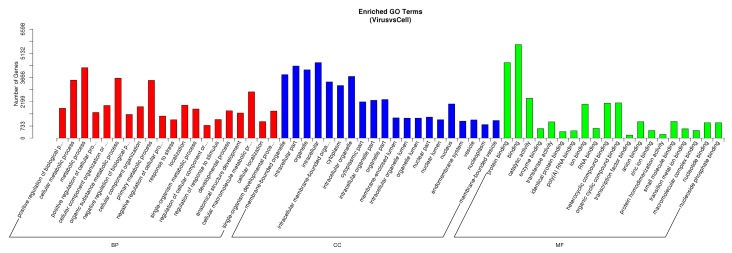
GO functional classification of all DE miRNA target genes. The GO distribution of all DE miRNA targets in the PCV2-infected cells versus mock-infected were classified into three categories: Biological process, cellular components, and molecular function.

**Figure 7:**
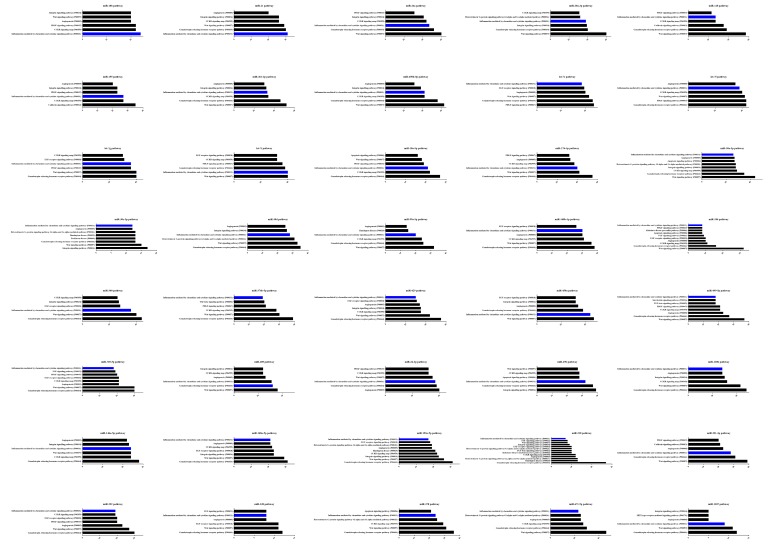
KEGG enrichment of all DE miRNA target genes. The KEGG enrichment analysis of DE miRNA targets in the PCV2-infected cells versus mock-infected showed that the pathways viz. inflammation mediated by chemokine and cytokine signaling pathway (highlight in blue), Wnt signaling pathway, CCKR signaling map governed by DE miRNAs were more perturbed in infected PK-15 than in mock control.

We predicted DE miRNA targets by using miRecords and found thousands of target genes for each DE miRNA. The GO and KEGG pathway analyses of each DE miRNA target gene showed that the annotated targets were mainly involved in immune system processes, metabolic process, binding, organelle, and other cell processes (Figure 6). The most frequently represented category of pathway was inflammation mediated by chemokine and cytokine signaling pathway (P00031) (Figure 7).

### 3.5. Construction of the miRNA-mRNA Network

**Figure 8:**
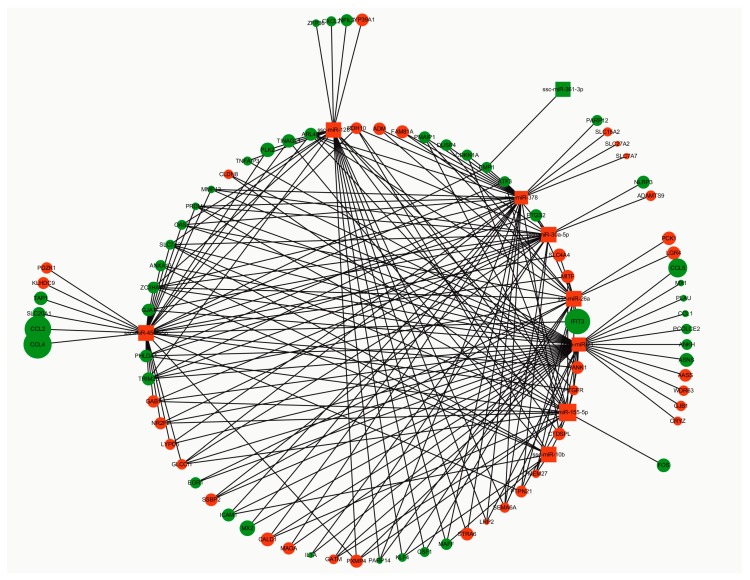
DE miRNA-mRNA network. The node color represents the up-regulation in red or down-regulation in green of miRNAs/mRNAs. The node size changes gradually from small to large in ascending order according to the log2 (fold change) of miRNAs or mRNAs. The box represents miRNA and the circle represents mRNA.

We predicted DE miRNA targets by using miRecords and found thousands of target genes for each DE miRNA. A total of 159 DE genes were identified by using GEO2R and were converted from IDs into official gene symbol by using DAVID 6.8 [31]. By intersecting the predicted target genes and DE genes, we identified many targets genes for each DE miRNA that exert momentous roles in PCV2 infection. The DE miRNA-mRNA network was constructed and visualized using Cytoscape. We constructed an miRNA-mRNA network, which provided a preliminary insight into the links between the DE miRNAs and mRNAs. As shown in Figure 8, nine miRNA nodes and 82 mRNA nodes composed the miRNA-mRNA network.

### 3.6. Dysregulated miRNA Expression Influences PCV2-Triggered Cellular Inflammatory Response

PCV2 infections were found to alter cytokine expression patterns in our previous study. To further determine whether DE miRNAs are involved in the PCV2-mediated inflammatory process, the DE miRNA effects on the cytokine mRNA production were examined.

**Figure 9:**
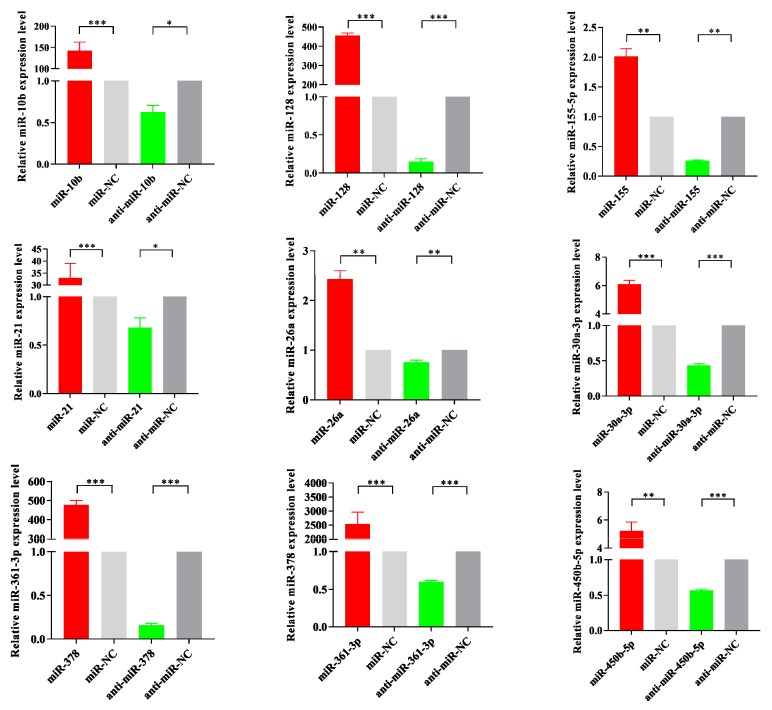
MiRNA mimics and inhibitors transfection effects in miRNA levels. PK-15 cells were transfected with miRNA mimics and inhibitors or control oligonucleotides for 24 h and then infected with PCV2 at a MOI of 10 for 36 h. The relative expression levels of nine miRNA were calculated using the 2^−ΔΔCT^ method and represented as the n-fold change compared to the control oligonucleotides sample. The U6 snRNA (small nuclear RNA) as an endogenous control. The data were expressed as mean ± standard error of measurement (SEM) from three independent experiments. Statistical significance was determined by Student’s t-test, with *p*-values of < 0.05 deemed to be statistically significant (*, *p* < 0.05; **, *p* < 0.01; ***, *p* < 0.001).

**Figure 10:**
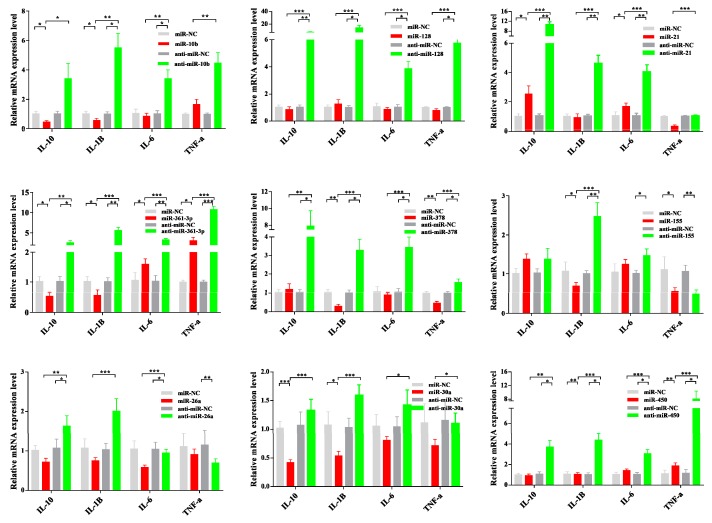
MiRNA mimics and inhibitors transfection effects in the TNF-α, IL-6, IL-1β, and IL-10 mRNA levels. PK-15 cells were transfected with miRNA mimics and inhibitors or control oligonucleotides for 24 h and then infected with PCV2 at a MOI of 10 for 36 h. The relative expression level of TNF-α, IL-6, IL-1β, and IL-10 were calculated using the 2^−ΔΔCT^ method and represented as the n-fold change compared to the control oligonucleotides sample. The GAPDH (glyceraldehyde-3-phosphate dehydrogenase) gene as an endogenous control. The data were expressed as mean ± standard error of measurement (SEM) from three independent experiments. Statistical significance was determined by Student’s t-test, with *p*-values of < 0.05 deemed to be statistically significant (*, *p* < 0.05; **, *p* < 0.01; ***, *p* < 0.001).

MiRNA mimics are double-stranded RNAs synthesized to simulate naturally occurring mature miRNAs, whereas inhibitors are chemically modified antisense single-stranded RNAs that curb the yielding of endogenous miRNAs by sequence complementarity. As shown in Figure 9, miRNA mimics transfection increased miRNA levels significantly in PCV2-infected cells, whereas inhibitors transfection diminished its levels. Interestingly, PCV2-induced inflammation cytokines, such as IL-6, IL-1β, TNF-α, and IL-10, were found to be significantly decreased after miRNAs were overexpressed. In contrast, the inhibition of endogenous miRNA significantly increased cytokine mRNA production (Figure 10).

## 4. Discussion

PCV2 pathogenicity depends on its ability to destroy the host immune system. Our previous studies have reported a correlation between a deregulated immune-inflammatory response and cytokine dysfunction in infected pigs and cells [6]. However, the mechanisms underlying PCV2 interaction with host immunity remain for further study. Previous studies have investigated mRNA and miRNA expression changes caused by PCV2 infection [15,16,17], but miRNA-mRNA interactions remain to be analyzed. In agreement with previous results, in this study, we utilized deep sequencing to discover small RNA expression profiling, evaluated the PCV2 encoded miRNA capability, and constructed the DE miRNA-mRNA network.

In this study, approximately 10 million 18–35 nt sRNA reads were generated by RNA sequencing. More than 297 mature miRNAs and 427 novel miRNAs candidates were identified, of which 44 were significantly differentially expressed. The sequencing was deeper and the analysis more accurate than sequencing results in previous studies, so we can utilize these data to execute more research. The KEGG pathway analysis revealed a significant enrichment for inflammation mediated by chemokine and cytokine signaling pathway in the PCV2 regulated miRNA targets. These results are consistent with our previous study on the transcription and proteomic analysis of PCV2-infected porcine alveolar macrophages (PAMs) [6,32]. Furthermore, these results indicated that PCV2 may regulate the immune-inflammatory response and cytokine dysfunction through miRNA-mRNA interaction.

Previous studies have tried to find PCV2-encoded miRNAs, but no small RNAs were found to match the PCV2 genome sequence with 100% homology. In this study, in order to eliminate the PCV2-encoded miRNAs effects on PCV2-induced inflammation, we also evaluated the capability of the PCV2 genome to encode miRNAs. Small RNAs with more than 10 times more data than previous studies were used for sequence alignment, and numerous PCV2 genome sequences from different genotypes were used to predict PCV2 miRNAs. Some small RNAs matched with the PCV2 genome at the origin of replication were found and many pre-miRNAs were predicted, but their expression was not detected, or their expression levels were not different between infected cells and non-infected cells. The locations of small RNAs matched with the PCV2 genome are unable to form a stem-loop structure. Hence, these small sequences are not the PCV2 encoded miRNA, but may be by-product of PCV2 Rep replication or transcription, or miRNA-like molecules. This result is consistent with previous studies. Hence, PCV2 may only regulate inflammatory responses by regulating cellular miRNAs.

Specific immunomodulatory miRNAs preferentially inhibit translation of many cellular anti-inflammatory proteins, and could drive a pro-inflammatory response in host cells [33]. Most of the 44 PCV2-regulated DE miRNAs are associated with inflammatory responses and cytokine regulation in many diseases. MiR-21 is one of the most highly expressed members of the miRNA family in many mammalian cell types. MiR-21 has emerged as a key mediator of the anti-inflammatory response in macrophages. At the same time, miR-21 has been shown to promote inflammatory mediators in non-hematopoietic cells resulting in neoplastic transformation [34]. Let-7e modulates the inflammatory response in vascular endothelial cells through ceRNA crosstalk [35]. MiR-10a-5p down-regulation in synoviocytes contributes to TBX5-controlled joint inflammation [36]. Extracellular microRNA-21 and microRNA-26a increase in the body fluids of rats with antigen-induced pulmonary inflammation [37]. MicroRNA-27b could modulate the inflammatory response and apoptosis during mycobacterium tuberculosis infection [38]. MiR-30a could remodel subcutaneous adipose tissue inflammation to improve insulin sensitivity in obesity and positively regulates the inflammatory response of microglia in experimental autoimmune encephalomyelitis [39,40]. MiR-30c-5p could regulate macrophage-mediated inflammation and pro-atherosclerosis pathways [41]. NF-κB-regulated miR-99a could modulate endothelial cell inflammation [42]. MiR-128 could regulate genes associated with inflammation and fibrosis of rat kidney cells in vitro [43]. MiR-429 possesses pro-inflammatory activities and may be a potential therapy target for LPS-induced lung injury [44]. MiR-29b could aggravate LPS-induced endothelial cell inflammatory damage by regulation of the NF-κB and JNK signaling pathways [45]. MiR-130b could attenuate vascular inflammation via negatively regulating tumor progression locus 2 (Tpl2) expression to promote obesity associated adipose tissue inflammation and insulin resistance in diabetic mice [46,47]. MiR-378 could promote hepatic inflammation and fibrosis via modulation of the NF-κB-TNFα pathway [48]. In this study, we have identified many overlapping genes between DE miRNA targets and DE mRNAs, and constructed the miRNA-mRNA network to visualize the interaction between DE miRNAs and mRNAs. Furthermore, we determined the DE miRNA functions in PCV2-mediated inflammation by assessing the effects of miRNA dysregulation on cytokine mRNA production. The results clearly indicated that miRNA dysregulation could affect PCV2 infection-induced cellular inflammatory responses.

The present study provides an insight into the underlying molecular interactions in miRNA-mRNA levels and gains some understanding of signaling pathways or star molecules that may be applied to PCV2 clear prevention. The function study of DE miRNAs, especially the star molecules could provide an important direction for further research on PCV2 pathogenesis. The further assessment of PCV2’s capacity to encode viral miRNAs could provide some ideas for viral miRNA discovery.

## 5. Conclusions

This is the first study to construct the putative interaction network of PCV2 infection caused by differently expressed miRNAs and mRNAs. The small RNA-seq showed that the expression of 44 cellular miRNAs was altered between PCV2-infected cells and non-infected cells. The pathways viz. inflammation mediated by chemokine and cytokine signaling pathway (P00031), was more perturbed in PCV2-infected cells than in the mock control. PCV2 could regulate the cellular inflammatory response through dysregulation of host miRNA rather than through viral miRNAs.

## Supplementary Materials

The following are available online at https://www.mdpi.com/1999-4915/11/11/1055/s1, Table S1: The sequences of the mimics, inhibitors, or scrambled oligonucleotides used to up/down regulate miRNA. Table S2: Oligonucleotides used as primers for miRNA-specific and mRNA qRT-PCR analysis. Table S3: Summary of deep sequencing data in mock- and PCV2-infected PK-15 cells. Table S4: Distribution of small RNA on the reference sequence. Table S5: SRNA classification and annotation statistics. Table S6: Expression of known matures miRNA in each sample. Table S7: Expression of novel matures miRNA in each sample. Table S8: Differentially expressed miRNAs between the PCV2-infected and mock-infected PK-15 cells. Table S9: The details of the small RNAs matched with the PCV2 genome. Table S10: Sequences and structure diagrams of miRNA-like hairpins predicted by using miREval. Table S11: The detailed information of each pre-miRNA.
