# Supplementary material for: PCV2 Regulates Cellular Inflammatory Responses through Dysregulating Cellular miRNA-mRNA Networks
Source: Viruses. 2019 Nov 13;11(11):1055. doi: 10.3390/v11111055 (PMC6893612; doi:10.3390/v11111055)
Supplement: Supplementary file 1 [file viruses-11-01055-s001.zip › Supplementary Files/Supplementary Table S1.docx]

**Supplementary Table S1.**

**The sequences of the mimics, inhibitors, or scrambled oligonucleotides used to up/down regulate miRNA.**

|  | **sequences** | |
| --- | --- | --- |
|  | sense（5'-3'） | antisense（5'-3'） |
| **ssc-miR-10b mimic** | UACCCUGUAGAACCGAAUUUGU | AAAUUCGGUUCUACAGGGUAUU |
| **ssc-miR-10b inhibitor** | ACAAAUUCGGUUCUACAGGGUA |  |
| **ssc-miR-128 mimic** | UCACAGUGAACCGGUCUCUUU | AGAGACCGGUUCACUGUGAUU |
| **ssc-miR-128 inhibitor** | AAAGAGACCGGUUCACUGUGA |  |
| **ssc-miR-21 mimic** | UAGCUUAUCAGACUGAUGUUGA | AACAUCAGUCUGAUAAGCUAUU |
| **ssc-miR-21 inhibitor** | UCAACAUCAGUCUGAUAAGCUA |  |
| **ssc-miR-26a mimic** | UUCAAGUAAUCCAGGAUAGGCU | CCUAUCCUGGAUUACUUGAAUU |
| **ssc-miR-26a inhibitor** | AGCCUAUCCUGGAUUACUUGAA |  |
| **ssc-miR-30a-3p mimic** | CUUUCAGUCGGAUGUUUGCAGC | UGCAAACAUCCGACUGAAAGUU |
| **ssc-miR-30a-3p inhibitor** | GCUGCAAACAUCCGACUGAAAG |  |
| **ssc-miR- 155-5p mimics** | UUAAUGCUAAUCGUGAUAGGGGU | CCCUAUCACGAUUAGCAUUAAUU |
| **ssc-miR-155-5p inhibitor** | ACCCCUAUCACGAUUAGCAUUAA |  |
| **ssc-miR-361-3p mimic** | UUAAUGCUAAUCGUGAUAGGGGU | CCCUAUCACGAUUAGCAUUAAUU |
| **ssc-miR-361-3p inhibitor** | ACCCCUAUCACGAUUAGCAUUAA |  |
| **ssc-miR-378 mimic** | ACUGGACUUGGAGUCAGAAGG | UUCUGACUCCAAGUCCAGUUU |
| **ssc-miR-378 inhibitor** | CCUUCUGACUCCAAGUCCAGU |  |
| **ssc-miR-450b-5p mimic** | UUUUGCAAUAUGUUCCUGAAUA | UUCAGGAACAUAUUGCAAAAUU |
| **ssc-miR-450b-5p inhibitor** | UAUUCAGGAACAUAUUGCAAAA |  |
| **miR-NC** | UUCUCCGAACGUGUCACGUTT | ACGUGACACGUUCGGAGAATT |
| **Anti-miR-NC** | CAGUACUUUUGUGUAGUACAA |  |
