# Supplementary material for: PCV2 Regulates Cellular Inflammatory Responses through Dysregulating Cellular miRNA-mRNA Networks
Source: Viruses. 2019 Nov 13;11(11):1055. doi: 10.3390/v11111055 (PMC6893612; doi:10.3390/v11111055)
Supplement: Supplementary file 1 [file viruses-11-01055-s001.zip › Supplementary Files/Supplementary Table S2.docx]

**Supplementary Table S2.**

**Oligonucleotides used as primers for miRNA-specific and mRNA qRT-PCR analysis**

| targets | name | sequence |
| --- | --- | --- |
| miR-10b | miR-10b-RT | GTCGTATCCAGTGCAGGGTCCGAGGTATTCGCACTGGATACGACACAAATTC |
|  | miR-10b-F | TGGTTG TACCCTGTAGAACCG |
| miR-146a-5p | miR-146a-5p-RT | GTCGTATCCAGTGCAGGGTCCGAGGTATTCGCACTGGATACGACAACCCATG |
|  | miR-146a-5p-F | TGGTTG TGAGAACTGAATTCCATG |
| miR-148b-3p | miR-148b-3p-RT | GTCGTATCCAGTGCAGGGTCCGAGGTATTCGCACTGGATACGACACAAAGTT |
|  | miR-148b-3p-F | TGGTTG TCAGTGCATCACAG |
| miR-210 | miR-210-RT | GTCGTATCCAGTGCAGGGTCCGAGGTATTCGCACTGGATACGACTCAGCCGC |
|  | miR-210-F | TGGTTG CTGTGCGTGTG |
| miR-30a-5p | miR-30a-5p-RT | GTCGTATCCAGTGCAGGGTCCGAGGTATTCGCACTGGATACGACCTTCCAGT |
|  | miR-30a-5p-F | TGGTTG TGTAAACATCCTCGAC |
| miR-30c-5p | miR-30c-5p-RT | GTCGTATCCAGTGCAGGGTCCGAGGTATTCGCACTGGATACGACGCTGAGAG |
|  | miR-30c-5p-F | TGGTTG TGTAAACATCCTACACTCT |
| miR-378 | miR-378-RT | GTCGTATCCAGTGCAGGGTCCGAGGTATTCGCACTGGATACGACGCCTTCTG |
|  | miR-378-F | TGGTTG ACTGGACTTGGAGTC |
| miR-450b-5p | miR-450b-5p-RT | GTCGTATCCAGTGCAGGGTCCGAGGTATTCGCACTGGATACGACTATTCAGG |
|  | miR-450b-5p-F | TGGTTG TTTTGCAATATGTTCCTGA |
| miR-21 | miR-21-RT | GTCGTATCCAGTGCAGGGTCCGAGGTATTCGCACTGGATACGACTCAACATC |
|  | miR-21-F | GAGACGTAGCTTATCAGACTGATG |
| miR-769-5p | miR-769-5p-RT | GTCGTATCCAGTGCAGGGTCCGAGGTATTCGCACTGGATACGACGCTCAGAA |
|  | miR-769-5p-F | TGGTTGTGAGACCTCTGGG |
| miR-128 | miR-128-RT | GTCGTATCCAGTGCAGGGTCCGAGGTATTCGCACTGGATACGACAAAGAGAC |
|  | miR-128-F | TGGTTGTCACAGTGAACCG |
| miR-30a-3p | miR-30a-3p-RT | GTCGTATCCAGTGCAGGGTCCGAGGTATTCGCACTGGATACGACGCTGCAAA |
|  | miR-30a-3p-F | TGGTTGCTTTCAGTCGGATG |
| miR-361-3p | miR-361-3p-RT | GTCGTATCCAGTGCAGGGTCCGAGGTATTCGCACTGGATACGACGCAAATCA |
|  | miR-361-3p-F | TGGTTGCCCCCAGGTG |
| miR-155 | miR-155-RT | GTCGTATCCAGTGCAGGGTCCGAGGTATTCGCACTGGATACGACCCCCTATC |
|  | miR-155-F | TGGTTGTTAATGCTAATTGTGATAGGG |
| miRNA universal reverse primer | UN-R | GTGCAGGGTCCGAGGT |
| U6 | U6-RT | CGCTTCACGAATTTGCGTGTC |
|  | U6-F | GCTTCGGCAGCACATATACT |
|  | U6-R | CTTCACGAATTTGCGTGTCAT |
| IL-6 | IL-6-F | GCATCACCTTTGGCATCTTCTTCC |
|  | IL-6-R | CCTTCAGTCCAGTCGCCTTCTCC |
| IL-1β | IL-1β-F | CTTGAAGAGAGAAGTGGTGTTCTG |
|  | IL-1β-R | ATCACACAAGACAGGTACAGATTCT |
| IL-10 | IL-10-F | GGTCAGCAACAAGTCGCCCATC |
|  | IL-10-R | GCATCCACTTCCCAACCAGCC |
| TNFα | TNFα-F | TGGTGGTGCCGACAGATG |
|  | TNFα-R | GGCTGATGGTGTGAGTGAGG |
| GAPDH | GAPDH-F | TGTGGGCATCAATGGATTTGG |
|  | GAPDH-R | ACACCATGTATTCAGGGTCCAT |
