# Supplementary material for: PCV2 Regulates Cellular Inflammatory Responses through Dysregulating Cellular miRNA-mRNA Networks
Source: Viruses. 2019 Nov 13;11(11):1055. doi: 10.3390/v11111055 (PMC6893612; doi:10.3390/v11111055)
Supplement: Supplementary file 1 [file viruses-11-01055-s001.zip › Supplementary Files/Supplementary Table S3.docx]

**Supplementary Table S3.**

**Summary of deep sequencing data in mock- and PCV2-infected PK-15 cells**

| **Sample** | **total reads** | **N% > 10%** | **low quality** | **5 adapter contamine** | **3 adapter null or insert null** | **with ployA/T/G/C** | **clean reads** |
| --- | --- | --- | --- | --- | --- | --- | --- |
| V_1 | 12821726 (100.00%) | 513 (0.00%) | 86499 (0.67%) | 1321 (0.01%) | 173532 (1.35%) | 28441 (0.22%) | 12531420 (97.74%) |
| V_2 | 14389763 (100.00%) | 553 (0.00%) | 100104 (0.70%) | 1497 (0.01%) | 220727 (1.53%) | 35213 (0.24%) | 14031669 (97.51%) |
| V_3 | 10960073 (100.00%) | 466 (0.00%) | 77305 (0.71%) | 1100 (0.01%) | 150230 (1.37%) | 27700 (0.25%) | 10703272 (97.66%) |
| C_1 | 14670375 (100.00%) | 491 (0.00%) | 78667 (0.54%) | 968 (0.01%) | 404138 (2.75%) | 15843 (0.11%) | 14170268 (96.59%) |
| C_2 | 11900506 (100.00%) | 395 (0.00%) | 66191 (0.56%) | 652 (0.01%) | 193702 (1.63%) | 10187 (0.09%) | 11629379 (97.72%) |
| C_3 | 12839196 (100.00%) | 385 (0.00%) | 71908 (0.56%) | 696 (0.01%) | 162799 (1.27%) | 11629 (0.09%) | 12591779 (98.07%) |
