# Supplementary material for: PCV2 Regulates Cellular Inflammatory Responses through Dysregulating Cellular miRNA-mRNA Networks
Source: Viruses. 2019 Nov 13;11(11):1055. doi: 10.3390/v11111055 (PMC6893612; doi:10.3390/v11111055)
Supplement: Supplementary file 1 [file viruses-11-01055-s001.zip › Supplementary Files/Supplementary Table S4.docx]

**Supplementary Table S4.**

**Distribution of small RNA on the reference sequence.**

| **Sample** | **Total sRNA** | **Mapped sRNA** | **"+" Mapped sRNA** | **"-" Mapped sRNA** |
| --- | --- | --- | --- | --- |
| V_3 | 9870721 (100.00%) | 8716711 (88.31%) | 6430109 (65.14%) | 2286602 (23.17%) |
| C_2 | 10941604 (100.00%) | 10005823 (91.45%) | 7440034 (68.00%) | 2565789 (23.45%) |
| C_3 | 11970634 (100.00%) | 10947313 (91.45%) | 8133660 (67.95%) | 2813653 (23.50%) |
| V_1 | 11438092 (100.00%) | 10162566 (88.85%) | 7529967 (65.83%) | 2632599 (23.02%) |
| C_1 | 13284791 (100.00%) | 11924927 (89.76%) | 8577429 (64.57%) | 3347498 (25.20%) |
| V_2 | 12636210 (100.00%) | 11194990 (88.59%) | 8324032 (65.87%) | 2870958 (22.72%) |
