# Supplementary material for: PCV2 Regulates Cellular Inflammatory Responses through Dysregulating Cellular miRNA-mRNA Networks
Source: Viruses. 2019 Nov 13;11(11):1055. doi: 10.3390/v11111055 (PMC6893612; doi:10.3390/v11111055)
Supplement: Supplementary file 1 [file viruses-11-01055-s001.zip › Supplementary Files/Supplementary Table S5.docx]

**Supplementary Table S5**

**SRNA classification and annotation statistics.**

| Types | C_1(percent) | C_2(percent) | C_3(percent) | V_1(percent) | V_2(percent) | V_3(percent) |
| --- | --- | --- | --- | --- | --- | --- |
| total | 100.00% | 100.00% | 100.00% | 100.00% | 100.00% | 100.00% |
| known_miRNA | 49.48% | 54.81% | 54.83% | 55.36% | 55.23% | 53.62% |
| rRNA | 0.25% | 0.24% | 0.25% | 0.20% | 0.23% | 0.23% |
| tRNA | 0.03% | 0.03% | 0.04% | 0.03% | 0.03% | 0.03% |
| snRNA | 0.09% | 0.09% | 0.09% | 0.08% | 0.09% | 0.08% |
| snoRNA | 1.61% | 1.44% | 1.62% | 1.46% | 1.68% | 1.73% |
| repeat | 1.80% | 1.58% | 1.69% | 1.37% | 1.51% | 1.49% |
| novel_miRNA | 0.40% | 0.50% | 0.51% | 0.42% | 0.41% | 0.39% |
| exon:+ | 1.30% | 1.08% | 1.13% | 0.99% | 1.15% | 1.17% |
| exon:- | 0.11% | 0.12% | 0.12% | 0.11% | 0.11% | 0.11% |
| intron:+ | 1.60% | 1.52% | 1.61% | 1.42% | 1.51% | 1.49% |
| intron:- | 0.34% | 0.28% | 0.30% | 0.23% | 0.23% | 0.22% |
| other | 42.98% | 38.30% | 37.82% | 38.33% | 37.80% | 39.43% |
