# Supplementary material for: PCV2 Regulates Cellular Inflammatory Responses through Dysregulating Cellular miRNA-mRNA Networks
Source: Viruses. 2019 Nov 13;11(11):1055. doi: 10.3390/v11111055 (PMC6893612; doi:10.3390/v11111055)
Supplement: Supplementary file 1 [file viruses-11-01055-s001.zip › Supplementary Files/Supplementary Table S6.docx]

**Supplementary Table S6.**

**Expression of known matures miRNA in each sample.**

| **miRNA** | **Counts** | | | | | |
| --- | --- | --- | --- | --- | --- | --- |
|  | **C_1** | **C_2** | **C_3** | **V_1** | **V_2** | **V_3** |
| ssc-let-7a | 39840 | 32571 | 36446 | 34518 | 35731 | 28759 |
| ssc-let-7c | 9076 | 7529 | 8153 | 7192 | 7760 | 6049 |
| ssc-let-7d-3p | 1500 | 1365 | 1507 | 1313 | 1512 | 1136 |
| ssc-let-7d-5p | 7278 | 5831 | 6475 | 5966 | 7060 | 5233 |
| ssc-let-7e | 7267 | 6825 | 7614 | 6454 | 6284 | 4959 |
| ssc-let-7f | 186089 | 169984 | 190401 | 164218 | 156478 | 128210 |
| ssc-let-7g | 158604 | 130456 | 142944 | 124919 | 127152 | 104631 |
| ssc-let-7i | 406689 | 349637 | 381998 | 372070 | 374649 | 309340 |
| ssc-miR-1 | 1004 | 922 | 1013 | 1037 | 1006 | 851 |
| ssc-miR-100 | 73875 | 92163 | 97278 | 93054 | 77108 | 58074 |
| ssc-miR-101 | 30097 | 32421 | 34456 | 33283 | 40119 | 29742 |
| ssc-miR-103 | 65228 | 60616 | 63889 | 62140 | 72459 | 53376 |
| ssc-miR-105-1 | 1 | 0 | 1 | 1 | 1 | 2 |
| ssc-miR-106a | 11 | 6 | 13 | 10 | 13 | 10 |
| ssc-miR-107 | 4531 | 3803 | 4039 | 3618 | 4301 | 3191 |
| ssc-miR-10a-3p | 1808 | 1461 | 1498 | 1507 | 1518 | 1241 |
| ssc-miR-10a-5p | 330347 | 322772 | 358377 | 286769 | 282523 | 226505 |
| ssc-miR-10b | 132217 | 126706 | 141638 | 107823 | 105912 | 88870 |
| ssc-miR-122 | 285 | 264 | 279 | 317 | 250 | 198 |
| ssc-miR-1224 | 0 | 0 | 0 | 0 | 1 | 0 |
| ssc-miR-124a | 27 | 30 | 31 | 34 | 49 | 33 |
| ssc-miR-125a | 3823 | 3147 | 3517 | 3376 | 3544 | 2834 |
| ssc-miR-125b | 3715 | 3434 | 3608 | 3683 | 3785 | 2941 |
| ssc-miR-126-3p | 195 | 225 | 246 | 251 | 260 | 201 |
| ssc-miR-126-5p | 43 | 31 | 40 | 37 | 68 | 35 |
| ssc-miR-127 | 15 | 19 | 29 | 30 | 32 | 27 |
| ssc-miR-1271 | 31 | 37 | 47 | 34 | 51 | 26 |
| ssc-miR-1277 | 4 | 5 | 4 | 7 | 7 | 6 |
| ssc-miR-128 | 11761 | 10679 | 11247 | 9472 | 9893 | 7211 |
| ssc-miR-1285 | 25 | 13 | 12 | 17 | 11 | 11 |
| ssc-miR-1296-5p | 270 | 239 | 304 | 277 | 266 | 217 |
| ssc-miR-129a-3p | 82 | 67 | 88 | 71 | 70 | 66 |
| ssc-miR-129a-5p | 1158 | 1147 | 1213 | 1233 | 1302 | 928 |
| ssc-miR-1306-3p | 136 | 119 | 142 | 141 | 158 | 111 |
| ssc-miR-1306-5p | 589 | 591 | 651 | 585 | 645 | 503 |
| ssc-miR-1307 | 4089 | 4687 | 4957 | 5337 | 6198 | 4429 |
| ssc-miR-130a | 98 | 66 | 88 | 79 | 86 | 57 |
| ssc-miR-130b | 130 | 117 | 121 | 146 | 209 | 138 |
| ssc-miR-132 | 36 | 51 | 75 | 52 | 57 | 44 |
| ssc-miR-133a-3p | 53 | 42 | 33 | 50 | 63 | 47 |
| ssc-miR-133a-5p | 7 | 8 | 1 | 17 | 17 | 5 |
| ssc-miR-133b | 8 | 3 | 0 | 3 | 3 | 3 |
| ssc-miR-1343 | 426 | 493 | 497 | 482 | 560 | 364 |
| ssc-miR-135 | 59 | 75 | 64 | 58 | 62 | 56 |
| ssc-miR-137 | 1 | 1 | 1 | 2 | 1 | 0 |
| ssc-miR-138 | 31 | 21 | 18 | 35 | 41 | 37 |
| ssc-miR-139-3p | 9 | 16 | 17 | 18 | 24 | 14 |
| ssc-miR-139-5p | 307 | 242 | 304 | 313 | 301 | 219 |
| ssc-miR-140-3p | 18001 | 18701 | 20535 | 18125 | 20734 | 14930 |
| ssc-miR-140-5p | 976 | 937 | 977 | 901 | 1078 | 776 |
| ssc-miR-142-3p | 97 | 63 | 76 | 96 | 94 | 87 |
| ssc-miR-142-5p | 33 | 38 | 43 | 52 | 48 | 38 |
| ssc-miR-143-3p | 26 | 17 | 29 | 23 | 7 | 16 |
| ssc-miR-144 | 0 | 3 | 0 | 0 | 0 | 0 |
| ssc-miR-145-3p | 3 | 1 | 0 | 2 | 5 | 1 |
| ssc-miR-145-5p | 17 | 15 | 24 | 27 | 28 | 19 |
| ssc-miR-1468 | 0 | 2 | 2 | 0 | 1 | 1 |
| ssc-miR-146a-5p | 1798 | 1326 | 1429 | 1966 | 2309 | 1839 |
| ssc-miR-146b | 157 | 122 | 132 | 139 | 165 | 143 |
| ssc-miR-148a-3p | 10535 | 12151 | 13248 | 13341 | 11550 | 9223 |
| ssc-miR-148a-5p | 76 | 105 | 81 | 120 | 159 | 102 |
| ssc-miR-148b-3p | 48027 | 46562 | 49839 | 38878 | 35616 | 28664 |
| ssc-miR-148b-5p | 159 | 151 | 179 | 178 | 214 | 150 |
| ssc-miR-149 | 267 | 280 | 265 | 309 | 351 | 246 |
| ssc-miR-150 | 0 | 0 | 0 | 1 | 2 | 2 |
| ssc-miR-151-3p | 25477 | 36659 | 39138 | 38461 | 36992 | 24597 |
| ssc-miR-151-5p | 611 | 585 | 549 | 565 | 518 | 411 |
| ssc-miR-152 | 7256 | 8518 | 8785 | 8744 | 10878 | 7210 |
| ssc-miR-153 | 1 | 1 | 2 | 2 | 4 | 1 |
| ssc-miR-155-3p | 3 | 5 | 4 | 6 | 6 | 1 |
| ssc-miR-155-5p | 6428 | 4812 | 5003 | 4684 | 5409 | 4698 |
| ssc-miR-15a | 232 | 199 | 193 | 182 | 254 | 186 |
| ssc-miR-15b | 1580 | 1609 | 1597 | 1389 | 1701 | 1119 |
| ssc-miR-16 | 3843 | 3944 | 4054 | 3501 | 4560 | 3487 |
| ssc-miR-17-3p | 65 | 37 | 68 | 67 | 95 | 62 |
| ssc-miR-17-5p | 16808 | 13942 | 14184 | 15729 | 20009 | 15136 |
| ssc-miR-181a | 1205 | 846 | 863 | 857 | 1135 | 846 |
| ssc-miR-181b | 1118 | 818 | 831 | 693 | 930 | 688 |
| ssc-miR-181c | 7 | 2 | 3 | 4 | 4 | 1 |
| ssc-miR-181d-5p | 86 | 61 | 81 | 65 | 77 | 61 |
| ssc-miR-182 | 52388 | 49074 | 56767 | 54122 | 52885 | 44611 |
| ssc-miR-183 | 29197 | 28773 | 31827 | 31812 | 31513 | 24779 |
| ssc-miR-1839-3p | 1 | 0 | 0 | 1 | 0 | 1 |
| ssc-miR-1839-5p | 120 | 118 | 139 | 133 | 144 | 103 |
| ssc-miR-184 | 3705 | 3684 | 3955 | 2991 | 2880 | 2288 |
| ssc-miR-185 | 9585 | 7547 | 8257 | 8338 | 10142 | 7523 |
| ssc-miR-186 | 10041 | 6579 | 7138 | 6985 | 8853 | 6732 |
| ssc-miR-187 | 65 | 96 | 86 | 88 | 82 | 57 |
| ssc-miR-18a | 1093 | 1194 | 1221 | 1261 | 1386 | 1125 |
| ssc-miR-18b | 3 | 1 | 3 | 3 | 3 | 2 |
| ssc-miR-190a | 320 | 234 | 231 | 220 | 284 | 192 |
| ssc-miR-190b | 8 | 2 | 7 | 8 | 13 | 6 |
| ssc-miR-191 | 16158 | 13638 | 14666 | 14742 | 17228 | 13355 |
| ssc-miR-192 | 32702 | 36487 | 39643 | 32672 | 36697 | 25602 |
| ssc-miR-193a-3p | 22 | 22 | 33 | 32 | 37 | 25 |
| ssc-miR-193a-5p | 670 | 661 | 748 | 843 | 912 | 669 |
| ssc-miR-194a | 12383 | 11156 | 11421 | 9354 | 13558 | 10140 |
| ssc-miR-194b-5p | 46 | 43 | 39 | 28 | 49 | 34 |
| ssc-miR-195 | 42 | 34 | 36 | 33 | 42 | 33 |
| ssc-miR-196a | 12 | 9 | 12 | 11 | 20 | 11 |
| ssc-miR-196b-3p | 17 | 11 | 14 | 19 | 23 | 18 |
| ssc-miR-196b-5p | 2178 | 2083 | 2328 | 1943 | 2034 | 1689 |
| ssc-miR-199a-3p | 17 | 11 | 19 | 15 | 14 | 15 |
| ssc-miR-199a-5p | 2 | 1 | 3 | 2 | 2 | 2 |
| ssc-miR-199b-3p | 17 | 11 | 19 | 15 | 14 | 15 |
| ssc-miR-199b-5p | 21 | 18 | 8 | 19 | 21 | 17 |
| ssc-miR-19a | 755 | 521 | 552 | 560 | 720 | 648 |
| ssc-miR-19b | 2807 | 2042 | 2184 | 2310 | 3391 | 2673 |
| ssc-miR-202-5p | 2 | 0 | 4 | 1 | 0 | 0 |
| ssc-miR-204 | 19 | 17 | 18 | 17 | 16 | 16 |
| ssc-miR-205 | 0 | 0 | 0 | 0 | 0 | 1 |
| ssc-miR-206 | 2 | 6 | 6 | 5 | 7 | 0 |
| ssc-miR-208b | 2 | 2 | 0 | 1 | 0 | 2 |
| ssc-miR-20a | 30732 | 26014 | 26917 | 26460 | 33666 | 25430 |
| ssc-miR-20b | 23 | 24 | 28 | 35 | 48 | 21 |
| ssc-miR-21 | 2365233 | 2281260 | 2497957 | 2582155 | 2975624 | 2158686 |
| ssc-miR-210 | 1979 | 1682 | 1583 | 2145 | 2703 | 2117 |
| ssc-miR-212 | 27 | 21 | 18 | 25 | 16 | 18 |
| ssc-miR-214 | 6 | 4 | 1 | 5 | 3 | 3 |
| ssc-miR-215 | 48 | 54 | 41 | 51 | 43 | 26 |
| ssc-miR-217 | 0 | 0 | 0 | 0 | 1 | 0 |
| ssc-miR-218-5p | 30 | 25 | 29 | 30 | 29 | 17 |
| ssc-miR-219a | 49 | 70 | 83 | 76 | 89 | 54 |
| ssc-miR-219b-3p | 7 | 6 | 10 | 7 | 5 | 7 |
| ssc-miR-221-3p | 33949 | 28726 | 28180 | 32965 | 42665 | 31398 |
| ssc-miR-221-5p | 5302 | 4273 | 4931 | 5038 | 5636 | 4653 |
| ssc-miR-222 | 21376 | 17979 | 19130 | 21323 | 24697 | 18878 |
| ssc-miR-22-3p | 1 | 0 | 2 | 0 | 1 | 0 |
| ssc-miR-224 | 21 | 35 | 24 | 32 | 82 | 35 |
| ssc-miR-22-5p | 0 | 0 | 0 | 0 | 1 | 1 |
| ssc-miR-2320-3p | 510 | 538 | 665 | 518 | 553 | 396 |
| ssc-miR-2320-5p | 1538 | 1429 | 1455 | 1251 | 1646 | 1274 |
| ssc-miR-2366 | 5 | 3 | 5 | 1 | 0 | 0 |
| ssc-miR-23a | 13796 | 12669 | 13110 | 12391 | 15200 | 11136 |
| ssc-miR-23b | 1568 | 1623 | 1566 | 1501 | 1810 | 1245 |
| ssc-miR-2411 | 292 | 315 | 382 | 374 | 356 | 282 |
| ssc-miR-24-1-5p | 2045 | 1806 | 1874 | 2064 | 2155 | 1821 |
| ssc-miR-24-2-5p | 29 | 26 | 34 | 39 | 45 | 34 |
| ssc-miR-24-3p | 13844 | 15438 | 16297 | 17911 | 21262 | 14168 |
| ssc-miR-2483 | 43 | 44 | 53 | 41 | 71 | 40 |
| ssc-miR-26a | 26898 | 22731 | 24951 | 19727 | 20212 | 15184 |
| ssc-miR-27a | 11485 | 14357 | 14785 | 14958 | 17027 | 10687 |
| ssc-miR-27b-3p | 51363 | 55123 | 58578 | 47589 | 51827 | 35147 |
| ssc-miR-27b-5p | 296 | 278 | 301 | 292 | 351 | 245 |
| ssc-miR-28-3p | 4774 | 5018 | 5459 | 4650 | 5007 | 3767 |
| ssc-miR-28-5p | 203 | 191 | 165 | 208 | 239 | 137 |
| ssc-miR-296-3p | 2319 | 2315 | 2440 | 2652 | 2915 | 2004 |
| ssc-miR-29a | 11586 | 9449 | 9857 | 10923 | 12744 | 9795 |
| ssc-miR-29b | 432 | 337 | 360 | 425 | 544 | 402 |
| ssc-miR-29c | 54 | 50 | 63 | 55 | 90 | 62 |
| ssc-miR-30a-3p | 10863 | 11869 | 12790 | 9983 | 9949 | 7214 |
| ssc-miR-30a-5p | 667134 | 564131 | 626812 | 466159 | 520840 | 406731 |
| ssc-miR-30b-3p | 84 | 62 | 76 | 109 | 90 | 55 |
| ssc-miR-30b-5p | 5477 | 4438 | 4676 | 4458 | 5024 | 4207 |
| ssc-miR-30c-1-3p | 95 | 78 | 114 | 112 | 108 | 74 |
| ssc-miR-30c-3p | 4597 | 4107 | 4486 | 3870 | 4064 | 3014 |
| ssc-miR-30c-5p | 30136 | 24598 | 26633 | 20073 | 22621 | 18480 |
| ssc-miR-30d | 136021 | 116802 | 131409 | 107917 | 118178 | 92865 |
| ssc-miR-30e-3p | 2595 | 2861 | 3116 | 2495 | 2553 | 1779 |
| ssc-miR-30e-5p | 22069 | 15984 | 17679 | 14980 | 18372 | 14837 |
| ssc-miR-31 | 3679 | 2907 | 2972 | 2912 | 3815 | 2826 |
| ssc-miR-32 | 1616 | 1686 | 1763 | 1822 | 1857 | 1364 |
| ssc-miR-320 | 4939 | 4302 | 4595 | 4826 | 6403 | 4888 |
| ssc-miR-324 | 79 | 68 | 53 | 50 | 64 | 48 |
| ssc-miR-325 | 0 | 0 | 1 | 0 | 0 | 0 |
| ssc-miR-326 | 0 | 0 | 1 | 0 | 2 | 0 |
| ssc-miR-328 | 328 | 321 | 336 | 354 | 355 | 198 |
| ssc-miR-331-3p | 423 | 432 | 439 | 472 | 559 | 385 |
| ssc-miR-331-5p | 144 | 198 | 201 | 196 | 194 | 148 |
| ssc-miR-335 | 1212 | 881 | 951 | 980 | 1281 | 1000 |
| ssc-miR-338 | 5 | 3 | 5 | 3 | 4 | 3 |
| ssc-miR-339-3p | 6 | 11 | 10 | 4 | 15 | 8 |
| ssc-miR-339-5p | 2564 | 2308 | 2313 | 2282 | 3022 | 2002 |
| ssc-miR-340 | 18720 | 15460 | 16581 | 13945 | 15900 | 11923 |
| ssc-miR-342 | 0 | 0 | 1 | 0 | 0 | 0 |
| ssc-miR-345-3p | 38 | 43 | 40 | 41 | 61 | 30 |
| ssc-miR-345-5p | 28 | 28 | 28 | 22 | 41 | 31 |
| ssc-miR-34a | 17 | 12 | 19 | 11 | 8 | 12 |
| ssc-miR-34c | 0 | 1 | 0 | 0 | 0 | 3 |
| ssc-miR-361-3p | 2905 | 2752 | 3054 | 2586 | 2729 | 2142 |
| ssc-miR-361-5p | 835 | 811 | 824 | 720 | 914 | 710 |
| ssc-miR-362 | 740 | 568 | 624 | 563 | 726 | 582 |
| ssc-miR-363 | 1 | 4 | 0 | 3 | 2 | 1 |
| ssc-miR-365-3p | 2250 | 1564 | 1723 | 1724 | 2050 | 1686 |
| ssc-miR-365-5p | 365 | 341 | 332 | 379 | 405 | 319 |
| ssc-miR-369 | 20 | 16 | 24 | 23 | 25 | 14 |
| ssc-miR-374a-3p | 4166 | 3862 | 4173 | 3270 | 3294 | 2521 |
| ssc-miR-374a-5p | 1993 | 2156 | 2092 | 1888 | 2236 | 1596 |
| ssc-miR-374b-3p | 505 | 450 | 570 | 446 | 436 | 357 |
| ssc-miR-374b-5p | 4237 | 4122 | 4389 | 3580 | 3880 | 2870 |
| ssc-miR-376a-3p | 0 | 0 | 1 | 0 | 1 | 0 |
| ssc-miR-376a-5p | 0 | 0 | 1 | 0 | 0 | 0 |
| ssc-miR-378 | 17939 | 22848 | 25071 | 28126 | 31217 | 21154 |
| ssc-miR-378b-3p | 1731 | 2012 | 2161 | 2578 | 2942 | 1944 |
| ssc-miR-381 | 0 | 0 | 0 | 1 | 0 | 2 |
| ssc-miR-382 | 26 | 30 | 29 | 39 | 32 | 25 |
| ssc-miR-383 | 50 | 52 | 44 | 49 | 68 | 36 |
| ssc-miR-421-3p | 316 | 314 | 315 | 269 | 338 | 249 |
| ssc-miR-421-5p | 4 | 5 | 1 | 1 | 0 | 1 |
| ssc-miR-423-3p | 31652 | 25410 | 27783 | 26359 | 30609 | 24094 |
| ssc-miR-423-5p | 11321 | 9832 | 10643 | 10799 | 11251 | 9201 |
| ssc-miR-424-5p | 182 | 181 | 185 | 161 | 186 | 116 |
| ssc-miR-425-3p | 338 | 409 | 457 | 389 | 538 | 317 |
| ssc-miR-425-5p | 7462 | 6333 | 6517 | 6960 | 7101 | 5857 |
| ssc-miR-429 | 8254 | 7579 | 8120 | 6743 | 7847 | 6047 |
| ssc-miR-432-5p | 1 | 1 | 0 | 0 | 0 | 0 |
| ssc-miR-4331 | 33 | 28 | 36 | 51 | 37 | 40 |
| ssc-miR-4332 | 1 | 1 | 1 | 3 | 0 | 0 |
| ssc-miR-4334-3p | 354 | 333 | 348 | 375 | 509 | 310 |
| ssc-miR-450a | 2901 | 2801 | 2989 | 2350 | 2501 | 1816 |
| ssc-miR-450b-3p | 7 | 10 | 14 | 11 | 8 | 6 |
| ssc-miR-450b-5p | 5682 | 5641 | 6173 | 4416 | 4607 | 3446 |
| ssc-miR-450c-3p | 44 | 35 | 48 | 28 | 30 | 29 |
| ssc-miR-450c-5p | 3873 | 4025 | 4464 | 3375 | 3633 | 2628 |
| ssc-miR-451 | 11 | 20 | 8 | 15 | 18 | 10 |
| ssc-miR-452 | 10 | 17 | 14 | 11 | 41 | 16 |
| ssc-miR-455-3p | 110 | 94 | 111 | 124 | 142 | 88 |
| ssc-miR-455-5p | 702 | 755 | 758 | 845 | 948 | 660 |
| ssc-miR-486 | 20 | 29 | 42 | 41 | 60 | 29 |
| ssc-miR-487b | 1 | 0 | 0 | 0 | 0 | 0 |
| ssc-miR-489 | 248 | 269 | 286 | 209 | 233 | 180 |
| ssc-miR-490-3p | 1710 | 1762 | 1927 | 1897 | 2049 | 1588 |
| ssc-miR-490-5p | 186 | 204 | 193 | 181 | 217 | 150 |
| ssc-miR-491 | 110 | 122 | 135 | 107 | 166 | 117 |
| ssc-miR-493-3p | 0 | 0 | 0 | 1 | 1 | 1 |
| ssc-miR-493-5p | 0 | 0 | 0 | 1 | 0 | 0 |
| ssc-miR-497 | 43 | 35 | 33 | 41 | 44 | 34 |
| ssc-miR-499-3p | 1 | 2 | 1 | 4 | 0 | 2 |
| ssc-miR-499-5p | 1034 | 828 | 912 | 789 | 852 | 604 |
| ssc-miR-500 | 1512 | 1463 | 1627 | 1465 | 1736 | 1306 |
| ssc-miR-504 | 70 | 100 | 97 | 61 | 85 | 56 |
| ssc-miR-505 | 1324 | 1052 | 1058 | 924 | 1095 | 896 |
| ssc-miR-532-3p | 368 | 301 | 304 | 246 | 367 | 293 |
| ssc-miR-532-5p | 17987 | 18586 | 19508 | 17293 | 17893 | 13492 |
| ssc-miR-542-3p | 4521 | 5030 | 5201 | 4607 | 5629 | 4252 |
| ssc-miR-542-5p | 14 | 15 | 16 | 18 | 16 | 8 |
| ssc-miR-545-3p | 26 | 27 | 22 | 18 | 20 | 17 |
| ssc-miR-545-5p | 2 | 0 | 1 | 1 | 7 | 3 |
| ssc-miR-551a | 13 | 19 | 18 | 14 | 17 | 7 |
| ssc-miR-574 | 282 | 307 | 352 | 301 | 419 | 271 |
| ssc-miR-582 | 255 | 309 | 318 | 303 | 351 | 259 |
| ssc-miR-615 | 33 | 35 | 49 | 31 | 33 | 27 |
| ssc-miR-628 | 64 | 53 | 73 | 52 | 57 | 41 |
| ssc-miR-652 | 97 | 107 | 138 | 91 | 112 | 63 |
| ssc-miR-664-3p | 76 | 78 | 74 | 60 | 63 | 53 |
| ssc-miR-664-5p | 34 | 28 | 34 | 17 | 42 | 23 |
| ssc-miR-671-3p | 132 | 168 | 186 | 180 | 196 | 124 |
| ssc-miR-671-5p | 237 | 220 | 220 | 249 | 329 | 251 |
| ssc-miR-676-3p | 0 | 0 | 0 | 0 | 1 | 0 |
| ssc-miR-6782-3p | 671 | 630 | 709 | 804 | 853 | 663 |
| ssc-miR-7 | 244472 | 189554 | 210710 | 179596 | 191663 | 163001 |
| ssc-miR-708-3p | 278 | 246 | 267 | 274 | 335 | 229 |
| ssc-miR-708-5p | 220 | 220 | 248 | 242 | 310 | 269 |
| ssc-miR-7134-3p | 1518 | 2113 | 2224 | 2128 | 2456 | 1701 |
| ssc-miR-7134-5p | 638 | 647 | 768 | 656 | 737 | 504 |
| ssc-miR-7135-3p | 17 | 19 | 19 | 31 | 25 | 21 |
| ssc-miR-7135-5p | 1 | 5 | 5 | 7 | 6 | 3 |
| ssc-miR-7136-5p | 0 | 1 | 0 | 1 | 0 | 1 |
| ssc-miR-7137-3p | 85 | 104 | 108 | 113 | 123 | 65 |
| ssc-miR-7137-5p | 332 | 267 | 252 | 259 | 329 | 219 |
| ssc-miR-7138-3p | 13 | 27 | 10 | 12 | 14 | 7 |
| ssc-miR-7138-5p | 84 | 43 | 73 | 61 | 55 | 40 |
| ssc-miR-7140-3p | 0 | 0 | 0 | 0 | 1 | 0 |
| ssc-miR-7141-5p | 0 | 0 | 0 | 2 | 0 | 0 |
| ssc-miR-7142-3p | 1095 | 1261 | 1482 | 1210 | 1270 | 884 |
| ssc-miR-7142-5p | 7 | 2 | 7 | 3 | 5 | 4 |
| ssc-miR-7143-3p | 1 | 1 | 1 | 1 | 2 | 0 |
| ssc-miR-7144-5p | 45 | 34 | 37 | 38 | 47 | 38 |
| ssc-miR-744 | 3132 | 3593 | 3601 | 3977 | 4418 | 3046 |
| ssc-miR-758 | 1 | 1 | 0 | 0 | 1 | 0 |
| ssc-miR-769-3p | 644 | 492 | 547 | 554 | 632 | 491 |
| ssc-miR-769-5p | 5703 | 5608 | 6251 | 5281 | 5742 | 4070 |
| ssc-miR-7857-3p | 3024 | 2543 | 2743 | 2656 | 3165 | 2405 |
| ssc-miR-874 | 1 | 2 | 1 | 2 | 1 | 0 |
| ssc-miR-885-3p | 37 | 33 | 21 | 31 | 30 | 20 |
| ssc-miR-885-5p | 89 | 94 | 91 | 62 | 67 | 59 |
| ssc-miR-9 | 128 | 121 | 130 | 118 | 131 | 82 |
| ssc-miR-9-1 | 128 | 121 | 130 | 118 | 131 | 82 |
| ssc-miR-92a | 63273 | 55235 | 59922 | 59937 | 59258 | 43693 |
| ssc-miR-92b-3p | 5343 | 5618 | 6090 | 5565 | 6108 | 4389 |
| ssc-miR-92b-5p | 544 | 444 | 522 | 458 | 465 | 394 |
| ssc-miR-935 | 35 | 22 | 36 | 29 | 33 | 24 |
| ssc-miR-95 | 79 | 85 | 95 | 90 | 105 | 66 |
| ssc-miR-96-5p | 4489 | 3407 | 3573 | 3889 | 4100 | 3438 |
| ssc-miR-9784-5p | 0 | 0 | 1 | 0 | 0 | 0 |
| ssc-miR-9785-5p | 9 | 8 | 3 | 4 | 3 | 2 |
| ssc-miR-9788-3p | 2 | 1 | 4 | 5 | 5 | 3 |
| ssc-miR-98 | 8765 | 7465 | 7742 | 7349 | 7574 | 6122 |
| ssc-miR-9810-3p | 15 | 13 | 18 | 16 | 17 | 8 |
| ssc-miR-9819-5p | 0 | 0 | 0 | 0 | 1 | 0 |
| ssc-miR-9820-5p | 1 | 0 | 1 | 1 | 0 | 2 |
| ssc-miR-9843-3p | 3947 | 4529 | 4883 | 4148 | 4619 | 3276 |
| ssc-miR-9851-3p | 9 | 11 | 12 | 12 | 11 | 4 |
| ssc-miR-9858-5p | 0 | 0 | 0 | 0 | 1 | 0 |
| ssc-miR-9860-5p | 15 | 20 | 6 | 14 | 25 | 10 |
| ssc-miR-99a | 28437 | 29908 | 30477 | 25257 | 25337 | 18217 |
| ssc-miR-99b | 22040 | 27633 | 29211 | 25391 | 24090 | 17233 |
