# Supplementary material for: PCV2 Regulates Cellular Inflammatory Responses through Dysregulating Cellular miRNA-mRNA Networks
Source: Viruses. 2019 Nov 13;11(11):1055. doi: 10.3390/v11111055 (PMC6893612; doi:10.3390/v11111055)
Supplement: Supplementary file 1 [file viruses-11-01055-s001.zip › Supplementary Files/Supplementary Table S7.docx]

**Additional file S7**

**Expression of novel matures miRNA in each sample.**

| **miRNA** | **counts** | | | | | |
| --- | --- | --- | --- | --- | --- | --- |
|  | **C_1** | **C_2** | **C_3** | **V_1** | **V_2** | **V_3** |
| **novel_1003** | **9** | **8** | **1** | **5** | **5** | **2** |
| **novel_1004** | **0** | **0** | **0** | **1** | **2** | **1** |
| **novel_1013** | **0** | **0** | **0** | **1** | **0** | **0** |
| **novel_1014** | **0** | **0** | **0** | **2** | **1** | **0** |
| **novel_1016** | **0** | **0** | **0** | **0** | **0** | **1** |
| **novel_1019** | **11** | **5** | **12** | **8** | **8** | **4** |
| **novel_1024** | **0** | **0** | **0** | **1** | **1** | **1** |
| **novel_1027** | **112** | **105** | **139** | **153** | **138** | **109** |
| **novel_1029** | **0** | **0** | **0** | **0** | **0** | **1** |
| **novel_1030** | **11** | **5** | **3** | **2** | **3** | **6** |
| **novel_1032** | **0** | **0** | **0** | **3** | **0** | **0** |
| **novel_1034** | **2** | **0** | **2** | **1** | **4** | **2** |
| **novel_1036** | **2** | **2** | **2** | **3** | **0** | **1** |
| **novel_1040** | **0** | **0** | **0** | **0** | **1** | **0** |
| **novel_1044** | **0** | **0** | **1** | **0** | **0** | **1** |
| **novel_1046** | **0** | **2** | **0** | **0** | **2** | **1** |
| **novel_1047** | **10** | **13** | **11** | **21** | **18** | **13** |
| **novel_1049** | **0** | **0** | **0** | **1** | **1** | **0** |
| **novel_1051** | **1** | **0** | **0** | **0** | **1** | **2** |
| **novel_1053** | **64** | **46** | **56** | **52** | **53** | **42** |
| **novel_1057** | **2** | **1** | **5** | **0** | **0** | **4** |
| **novel_1059** | **0** | **1** | **0** | **0** | **1** | **0** |
| **novel_106** | **554** | **557** | **592** | **559** | **579** | **479** |
| **novel_1062** | **2** | **1** | **4** | **0** | **0** | **2** |
| **novel_1063** | **0** | **4** | **4** | **6** | **0** | **0** |
| **novel_1065** | **1** | **4** | **3** | **1** | **4** | **9** |
| **novel_1071** | **0** | **0** | **1** | **0** | **0** | **0** |
| **novel_1081** | **2** | **1** | **4** | **0** | **1** | **2** |
| **novel_1082** | **0** | **4** | **1** | **1** | **0** | **0** |
| **novel_1083** | **0** | **0** | **0** | **1** | **1** | **3** |
| **novel_1085** | **1** | **1** | **0** | **2** | **1** | **1** |
| **novel_1089** | **1** | **1** | **3** | **1** | **1** | **0** |
| **novel_109** | **487** | **516** | **568** | **532** | **523** | **403** |
| **novel_1093** | **1** | **0** | **0** | **1** | **1** | **0** |
| **novel_1096** | **0** | **0** | **0** | **1** | **1** | **2** |
| **novel_1098** | **2** | **6** | **5** | **5** | **6** | **8** |
| **novel_110** | **37** | **20** | **13** | **18** | **26** | **19** |
| **novel_1100** | **0** | **0** | **0** | **3** | **3** | **1** |
| **novel_1101** | **0** | **2** | **0** | **0** | **0** | **2** |
| **novel_1103** | **0** | **2** | **1** | **3** | **1** | **0** |
| **novel_1104** | **0** | **0** | **0** | **1** | **0** | **0** |
| **novel_1107** | **8** | **13** | **18** | **22** | **14** | **8** |
| **novel_1108** | **1** | **0** | **0** | **0** | **0** | **1** |
| **novel_1109** | **3** | **1** | **0** | **0** | **0** | **0** |
| **novel_1110** | **0** | **0** | **0** | **1** | **1** | **0** |
| **novel_112** | **480** | **472** | **463** | **500** | **530** | **428** |
| **novel_1127** | **1** | **1** | **0** | **2** | **2** | **1** |
| **novel_1128** | **9** | **13** | **14** | **10** | **6** | **9** |
| **novel_1129** | **0** | **0** | **0** | **1** | **0** | **0** |
| **novel_1130** | **0** | **0** | **0** | **1** | **2** | **0** |
| **novel_1131** | **0** | **0** | **4** | **2** | **0** | **0** |
| **novel_1132** | **0** | **0** | **0** | **0** | **1** | **0** |
| **novel_1134** | **0** | **1** | **1** | **0** | **0** | **2** |
| **novel_1136** | **0** | **3** | **2** | **2** | **0** | **0** |
| **novel_1137** | **0** | **0** | **0** | **0** | **2** | **0** |
| **novel_1140** | **0** | **0** | **2** | **3** | **0** | **1** |
| **novel_1141** | **7** | **2** | **8** | **8** | **4** | **2** |
| **novel_1143** | **0** | **0** | **0** | **1** | **0** | **0** |
| **novel_1146** | **4** | **1** | **1** | **3** | **2** | **2** |
| **novel_1155** | **0** | **0** | **0** | **1** | **4** | **1** |
| **novel_1160** | **0** | **0** | **0** | **0** | **2** | **0** |
| **novel_1162** | **0** | **0** | **0** | **2** | **0** | **1** |
| **novel_1164** | **1** | **1** | **0** | **0** | **0** | **0** |
| **novel_1168** | **0** | **1** | **0** | **0** | **0** | **0** |
| **novel_1169** | **0** | **3** | **1** | **0** | **0** | **0** |
| **novel_1170** | **19** | **25** | **27** | **22** | **16** | **21** |
| **novel_1172** | **4** | **8** | **2** | **2** | **3** | **2** |
| **novel_1173** | **1** | **0** | **1** | **1** | **0** | **0** |
| **novel_1180** | **0** | **0** | **0** | **8** | **14** | **6** |
| **novel_1181** | **0** | **1** | **0** | **3** | **0** | **0** |
| **novel_1186** | **0** | **0** | **2** | **0** | **4** | **1** |
| **novel_1188** | **19** | **21** | **18** | **20** | **27** | **25** |
| **novel_1191** | **0** | **0** | **2** | **0** | **1** | **0** |
| **novel_1193** | **1** | **0** | **0** | **2** | **0** | **0** |
| **novel_1195** | **23** | **20** | **26** | **20** | **16** | **14** |
| **novel_1197** | **1** | **0** | **1** | **2** | **0** | **0** |
| **novel_1199** | **23** | **18** | **25** | **26** | **23** | **13** |
| **novel_120** | **161** | **166** | **190** | **190** | **210** | **174** |
| **novel_1201** | **0** | **0** | **1** | **0** | **0** | **0** |
| **novel_1209** | **3** | **0** | **0** | **0** | **0** | **0** |
| **novel_1210** | **3** | **1** | **0** | **0** | **2** | **0** |
| **novel_1213** | **0** | **0** | **3** | **1** | **2** | **0** |
| **novel_1214** | **0** | **0** | **1** | **0** | **2** | **0** |
| **novel_1217** | **9** | **7** | **8** | **7** | **17** | **6** |
| **novel_1218** | **0** | **0** | **1** | **0** | **1** | **0** |
| **novel_1221** | **3** | **0** | **1** | **1** | **0** | **0** |
| **novel_1222** | **1** | **0** | **0** | **1** | **0** | **0** |
| **novel_1223** | **0** | **0** | **0** | **1** | **0** | **0** |
| **novel_1226** | **1** | **3** | **3** | **0** | **1** | **0** |
| **novel_1229** | **0** | **0** | **0** | **2** | **1** | **2** |
| **novel_123** | **267** | **233** | **310** | **244** | **314** | **227** |
| **novel_1230** | **1** | **0** | **0** | **0** | **0** | **0** |
| **novel_1233** | **0** | **1** | **2** | **0** | **0** | **0** |
| **novel_1234** | **1** | **1** | **2** | **0** | **1** | **0** |
| **novel_1235** | **0** | **0** | **0** | **2** | **1** | **1** |
| **novel_1239** | **0** | **0** | **2** | **0** | **1** | **2** |
| **novel_1240** | **0** | **0** | **0** | **1** | **3** | **2** |
| **novel_1241** | **2** | **0** | **1** | **1** | **1** | **0** |
| **novel_126** | **256** | **215** | **211** | **191** | **208** | **138** |
| **novel_133** | **88** | **101** | **76** | **96** | **116** | **72** |
| **novel_142** | **3** | **2** | **0** | **0** | **0** | **1** |
| **novel_144** | **198** | **120** | **121** | **116** | **134** | **129** |
| **novel_151** | **56** | **44** | **36** | **53** | **74** | **40** |
| **novel_153** | **114** | **91** | **127** | **105** | **93** | **80** |
| **novel_155** | **135** | **131** | **121** | **107** | **119** | **77** |
| **novel_156** | **59** | **47** | **38** | **71** | **57** | **42** |
| **novel_162** | **101** | **77** | **76** | **77** | **72** | **50** |
| **novel_167** | **65** | **66** | **72** | **69** | **68** | **57** |
| **novel_168** | **11** | **10** | **15** | **17** | **16** | **8** |
| **novel_173** | **39** | **28** | **34** | **35** | **43** | **43** |
| **novel_184** | **20** | **33** | **33** | **35** | **39** | **32** |
| **novel_188** | **8** | **17** | **4** | **14** | **12** | **9** |
| **novel_189** | **12** | **28** | **39** | **31** | **26** | **19** |
| **novel_193** | **43** | **31** | **26** | **36** | **46** | **20** |
| **novel_194** | **4** | **5** | **2** | **1** | **2** | **0** |
| **novel_197** | **33** | **32** | **20** | **26** | **52** | **28** |
| **novel_198** | **22** | **35** | **22** | **34** | **34** | **29** |
| **novel_203** | **3** | **1** | **5** | **3** | **5** | **6** |
| **novel_204** | **44** | **23** | **26** | **27** | **22** | **18** |
| **novel_206** | **10** | **17** | **16** | **14** | **16** | **11** |
| **novel_207** | **22** | **35** | **19** | **30** | **30** | **26** |
| **novel_220** | **13** | **14** | **23** | **19** | **19** | **8** |
| **novel_224** | **16** | **16** | **25** | **13** | **29** | **19** |
| **novel_227** | **22** | **19** | **16** | **15** | **16** | **17** |
| **novel_232** | **12** | **4** | **13** | **14** | **11** | **19** |
| **novel_236** | **6** | **9** | **12** | **13** | **9** | **11** |
| **novel_237** | **17** | **16** | **16** | **12** | **15** | **14** |
| **novel_240** | **4** | **4** | **5** | **13** | **2** | **3** |
| **novel_242** | **17** | **12** | **12** | **17** | **6** | **9** |
| **novel_250** | **1** | **0** | **1** | **0** | **1** | **0** |
| **novel_254** | **3** | **0** | **0** | **0** | **0** | **2** |
| **novel_262** | **3** | **10** | **16** | **11** | **10** | **7** |
| **novel_264** | **6** | **10** | **11** | **10** | **10** | **4** |
| **novel_265** | **3** | **7** | **5** | **9** | **7** | **7** |
| **novel_266** | **6** | **11** | **2** | **12** | **8** | **4** |
| **novel_270** | **14** | **4** | **9** | **5** | **9** | **5** |
| **novel_274** | **4** | **6** | **8** | **9** | **7** | **5** |
| **novel_275** | **7** | **17** | **6** | **6** | **10** | **2** |
| **novel_276** | **13** | **10** | **13** | **17** | **8** | **8** |
| **novel_277** | **4** | **14** | **14** | **13** | **10** | **6** |
| **novel_279** | **5** | **4** | **1** | **3** | **8** | **5** |
| **novel_280** | **12** | **10** | **9** | **6** | **9** | **6** |
| **novel_287** | **8** | **2** | **4** | **5** | **3** | **2** |
| **novel_294** | **7** | **5** | **12** | **8** | **10** | **8** |
| **novel_298** | **4** | **7** | **5** | **2** | **11** | **10** |
| **novel_299** | **7** | **7** | **10** | **14** | **10** | **10** |
| **novel_304** | **10** | **5** | **5** | **2** | **1** | **2** |
| **novel_307** | **2** | **4** | **2** | **1** | **5** | **0** |
| **novel_308** | **2** | **2** | **1** | **1** | **2** | **4** |
| **novel_309** | **1** | **5** | **3** | **6** | **5** | **1** |
| **novel_311** | **3** | **2** | **6** | **9** | **3** | **3** |
| **novel_313** | **2** | **0** | **3** | **0** | **0** | **0** |
| **novel_316** | **3** | **3** | **3** | **7** | **5** | **1** |
| **novel_317** | **16** | **3** | **7** | **7** | **11** | **6** |
| **novel_323** | **11** | **5** | **9** | **8** | **4** | **5** |
| **novel_327** | **11** | **7** | **9** | **3** | **4** | **7** |
| **novel_328** | **7** | **3** | **3** | **4** | **6** | **3** |
| **novel_330** | **6** | **9** | **13** | **7** | **11** | **9** |
| **novel_332** | **0** | **0** | **1** | **0** | **0** | **0** |
| **novel_334** | **7** | **4** | **6** | **3** | **4** | **6** |
| **novel_338** | **3** | **5** | **9** | **1** | **6** | **3** |
| **novel_345** | **3** | **4** | **3** | **2** | **5** | **7** |
| **novel_347** | **3** | **6** | **5** | **4** | **3** | **2** |
| **novel_349** | **3** | **3** | **6** | **4** | **5** | **4** |
| **novel_350** | **4** | **7** | **2** | **2** | **2** | **4** |
| **novel_351** | **4** | **3** | **5** | **8** | **5** | **4** |
| **novel_352** | **1** | **0** | **1** | **0** | **0** | **0** |
| **novel_355** | **0** | **1** | **1** | **0** | **0** | **0** |
| **novel_356** | **0** | **0** | **0** | **1** | **0** | **0** |
| **novel_359** | **3** | **3** | **4** | **2** | **5** | **5** |
| **novel_362** | **5** | **2** | **4** | **3** | **1** | **5** |
| **novel_363** | **1** | **4** | **1** | **3** | **2** | **0** |
| **novel_367** | **2** | **0** | **2** | **1** | **0** | **5** |
| **novel_369** | **3** | **4** | **3** | **8** | **7** | **1** |
| **novel_370** | **5** | **3** | **4** | **5** | **4** | **5** |
| **novel_383** | **0** | **2** | **1** | **1** | **2** | **1** |
| **novel_384** | **1** | **4** | **1** | **3** | **5** | **1** |
| **novel_386** | **2** | **1** | **2** | **1** | **4** | **2** |
| **novel_388** | **3** | **4** | **4** | **4** | **3** | **1** |
| **novel_389** | **4** | **3** | **0** | **0** | **6** | **5** |
| **novel_390** | **2** | **4** | **3** | **2** | **6** | **2** |
| **novel_392** | **7** | **4** | **1** | **1** | **5** | **4** |
| **novel_394** | **22** | **17** | **20** | **12** | **23** | **18** |
| **novel_398** | **4** | **3** | **5** | **1** | **2** | **1** |
| **novel_399** | **0** | **0** | **5** | **2** | **2** | **1** |
| **novel_400** | **0** | **2** | **2** | **3** | **1** | **1** |
| **novel_401** | **1** | **0** | **1** | **2** | **0** | **2** |
| **novel_402** | **0** | **0** | **1** | **1** | **0** | **1** |
| **novel_406** | **0** | **0** | **3** | **2** | **1** | **3** |
| **novel_408** | **0** | **0** | **0** | **8** | **4** | **3** |
| **novel_411** | **5** | **4** | **3** | **3** | **3** | **1** |
| **novel_414** | **2** | **2** | **2** | **1** | **4** | **5** |
| **novel_416** | **1** | **3** | **2** | **3** | **1** | **2** |
| **novel_420** | **2** | **0** | **3** | **1** | **2** | **1** |
| **novel_421** | **3** | **1** | **4** | **1** | **1** | **1** |
| **novel_422** | **2** | **8** | **3** | **5** | **1** | **2** |
| **novel_424** | **0** | **1** | **1** | **1** | **3** | **0** |
| **novel_428** | **1** | **0** | **0** | **0** | **0** | **0** |
| **novel_429** | **1** | **2** | **0** | **1** | **1** | **0** |
| **novel_433** | **0** | **0** | **0** | **2** | **0** | **0** |
| **novel_436** | **2** | **3** | **0** | **0** | **1** | **1** |
| **novel_441** | **2** | **0** | **0** | **0** | **5** | **3** |
| **novel_442** | **1** | **2** | **1** | **3** | **0** | **0** |
| **novel_443** | **1** | **1** | **1** | **1** | **1** | **2** |
| **novel_446** | **1** | **2** | **0** | **2** | **1** | **0** |
| **novel_448** | **4** | **0** | **0** | **1** | **1** | **1** |
| **novel_451** | **2** | **3** | **0** | **1** | **2** | **0** |
| **novel_456** | **1** | **2** | **1** | **0** | **2** | **0** |
| **novel_457** | **0** | **2** | **0** | **0** | **0** | **0** |
| **novel_461** | **3** | **3** | **0** | **0** | **1** | **0** |
| **novel_464** | **0** | **0** | **0** | **0** | **3** | **0** |
| **novel_466** | **0** | **4** | **0** | **2** | **2** | **1** |
| **novel_468** | **0** | **0** | **1** | **3** | **0** | **1** |
| **novel_469** | **4** | **0** | **0** | **1** | **2** | **4** |
| **novel_473** | **0** | **3** | **2** | **0** | **1** | **2** |
| **novel_475** | **2** | **0** | **0** | **0** | **1** | **2** |
| **novel_478** | **0** | **1** | **0** | **0** | **0** | **0** |
| **novel_479** | **0** | **0** | **2** | **4** | **1** | **0** |
| **novel_480** | **1** | **0** | **1** | **2** | **1** | **1** |
| **novel_482** | **2** | **0** | **1** | **1** | **0** | **1** |
| **novel_491** | **1** | **2** | **0** | **2** | **2** | **1** |
| **novel_493** | **0** | **2** | **0** | **1** | **0** | **1** |
| **novel_496** | **0** | **1** | **1** | **0** | **0** | **0** |
| **novel_497** | **1** | **0** | **1** | **4** | **0** | **0** |
| **novel_498** | **0** | **0** | **0** | **3** | **0** | **0** |
| **novel_501** | **0** | **1** | **0** | **2** | **0** | **3** |
| **novel_504** | **0** | **0** | **1** | **0** | **0** | **0** |
| **novel_505** | **1** | **3** | **1** | **0** | **0** | **0** |
| **novel_506** | **0** | **0** | **1** | **0** | **0** | **0** |
| **novel_510** | **4** | **3** | **0** | **1** | **1** | **0** |
| **novel_519** | **2** | **0** | **1** | **2** | **4** | **1** |
| **novel_520** | **0** | **1** | **2** | **0** | **3** | **3** |
| **novel_521** | **0** | **0** | **0** | **0** | **0** | **1** |
| **novel_522** | **0** | **2** | **0** | **0** | **0** | **0** |
| **novel_524** | **0** | **1** | **0** | **0** | **0** | **0** |
| **novel_527** | **0** | **0** | **0** | **0** | **0** | **1** |
| **novel_529** | **0** | **0** | **0** | **0** | **0** | **1** |
| **novel_532** | **0** | **0** | **2** | **1** | **1** | **0** |
| **novel_533** | **0** | **0** | **1** | **0** | **0** | **0** |
| **novel_535** | **1** | **1** | **2** | **1** | **0** | **3** |
| **novel_536** | **0** | **1** | **0** | **1** | **1** | **1** |
| **novel_540** | **1** | **1** | **2** | **2** | **0** | **0** |
| **novel_542** | **0** | **0** | **0** | **2** | **0** | **0** |
| **novel_546** | **0** | **2** | **3** | **0** | **0** | **0** |
| **novel_548** | **2** | **2** | **0** | **1** | **2** | **1** |
| **novel_552** | **0** | **3** | **0** | **1** | **0** | **3** |
| **novel_554** | **0** | **0** | **1** | **0** | **0** | **1** |
| **novel_555** | **1** | **1** | **1** | **5** | **0** | **0** |
| **novel_557** | **0** | **0** | **0** | **1** | **2** | **1** |
| **novel_561** | **1** | **0** | **0** | **1** | **0** | **0** |
| **novel_563** | **0** | **0** | **0** | **0** | **1** | **0** |
| **novel_566** | **1** | **1** | **0** | **0** | **1** | **0** |
| **novel_567** | **1** | **2** | **4** | **0** | **1** | **0** |
| **novel_568** | **0** | **0** | **1** | **0** | **0** | **0** |
| **novel_569** | **1** | **0** | **2** | **2** | **1** | **1** |
| **novel_571** | **0** | **0** | **1** | **1** | **0** | **0** |
| **novel_582** | **2** | **1** | **0** | **0** | **0** | **1** |
| **novel_588** | **8** | **6** | **6** | **7** | **4** | **5** |
| **novel_590** | **3** | **0** | **1** | **3** | **5** | **0** |
| **novel_592** | **3** | **3** | **1** | **0** | **4** | **1** |
| **novel_593** | **0** | **0** | **0** | **0** | **1** | **0** |
| **novel_596** | **0** | **1** | **1** | **0** | **1** | **0** |
| **novel_597** | **0** | **2** | **2** | **3** | **1** | **0** |
| **novel_600** | **3** | **2** | **0** | **4** | **3** | **1** |
| **novel_603** | **1** | **0** | **0** | **0** | **0** | **0** |
| **novel_604** | **0** | **1** | **0** | **0** | **0** | **0** |
| **novel_605** | **1** | **0** | **0** | **0** | **0** | **0** |
| **novel_608** | **1** | **3** | **2** | **2** | **2** | **1** |
| **novel_609** | **0** | **2** | **1** | **1** | **3** | **0** |
| **novel_613** | **18** | **30** | **22** | **22** | **18** | **12** |
| **novel_614** | **0** | **2** | **4** | **0** | **1** | **0** |
| **novel_615** | **2** | **0** | **2** | **0** | **0** | **1** |
| **novel_619** | **1** | **1** | **1** | **3** | **2** | **1** |
| **novel_622** | **41** | **43** | **35** | **47** | **58** | **35** |
| **novel_624** | **0** | **1** | **2** | **0** | **0** | **2** |
| **novel_627** | **3** | **1** | **3** | **2** | **7** | **1** |
| **novel_630** | **1** | **0** | **1** | **1** | **0** | **2** |
| **novel_632** | **5** | **4** | **2** | **1** | **3** | **4** |
| **novel_633** | **3** | **5** | **10** | **7** | **5** | **3** |
| **novel_636** | **0** | **0** | **1** | **0** | **0** | **1** |
| **novel_637** | **1** | **1** | **2** | **1** | **0** | **2** |
| **novel_639** | **4** | **3** | **3** | **3** | **4** | **2** |
| **novel_640** | **1** | **1** | **4** | **2** | **1** | **4** |
| **novel_647** | **0** | **0** | **2** | **0** | **1** | **3** |
| **novel_649** | **6** | **13** | **9** | **11** | **6** | **7** |
| **novel_650** | **0** | **0** | **2** | **4** | **1** | **2** |
| **novel_652** | **1** | **1** | **2** | **0** | **0** | **3** |
| **novel_654** | **7** | **2** | **2** | **2** | **4** | **2** |
| **novel_656** | **1** | **0** | **2** | **2** | **2** | **1** |
| **novel_657** | **1** | **3** | **0** | **0** | **2** | **1** |
| **novel_663** | **0** | **2** | **1** | **2** | **1** | **2** |
| **novel_666** | **15** | **13** | **17** | **13** | **20** | **11** |
| **novel_667** | **4** | **4** | **8** | **9** | **15** | **7** |
| **novel_672** | **438** | **610** | **647** | **583** | **595** | **387** |
| **novel_673** | **6** | **5** | **7** | **4** | **6** | **0** |
| **novel_674** | **1** | **0** | **2** | **2** | **0** | **1** |
| **novel_682** | **4** | **1** | **1** | **1** | **2** | **0** |
| **novel_683** | **2** | **0** | **1** | **0** | **2** | **0** |
| **novel_687** | **2112** | **2414** | **2597** | **2642** | **2469** | **1814** |
| **novel_691** | **61** | **57** | **58** | **42** | **64** | **40** |
| **novel_692** | **7** | **2** | **5** | **2** | **4** | **3** |
| **novel_693** | **7** | **5** | **10** | **7** | **14** | **10** |
| **novel_694** | **4** | **2** | **3** | **4** | **2** | **0** |
| **novel_696** | **1** | **1** | **0** | **3** | **3** | **2** |
| **novel_698** | **27** | **24** | **25** | **15** | **14** | **21** |
| **novel_7** | **38773** | **40769** | **45361** | **32986** | **35992** | **26668** |
| **novel_700** | **0** | **2** | **0** | **3** | **1** | **0** |
| **novel_702** | **7** | **17** | **12** | **33** | **28** | **14** |
| **novel_705** | **21** | **17** | **22** | **25** | **18** | **29** |
| **novel_707** | **3** | **2** | **1** | **1** | **2** | **1** |
| **novel_709** | **3** | **3** | **4** | **3** | **0** | **2** |
| **novel_712** | **0** | **0** | **1** | **0** | **0** | **0** |
| **novel_713** | **0** | **0** | **1** | **0** | **0** | **0** |
| **novel_714** | **0** | **1** | **0** | **0** | **0** | **0** |
| **novel_715** | **1** | **0** | **0** | **0** | **0** | **1** |
| **novel_719** | **5** | **4** | **1** | **5** | **9** | **3** |
| **novel_720** | **2** | **3** | **3** | **9** | **9** | **2** |
| **novel_721** | **1** | **4** | **1** | **3** | **2** | **0** |
| **novel_723** | **2** | **0** | **0** | **0** | **4** | **2** |
| **novel_726** | **2** | **3** | **1** | **0** | **1** | **1** |
| **novel_730** | **0** | **2** | **0** | **0** | **0** | **1** |
| **novel_731** | **1** | **3** | **0** | **3** | **0** | **1** |
| **novel_732** | **1** | **0** | **1** | **1** | **1** | **0** |
| **novel_735** | **6** | **11** | **4** | **2** | **4** | **9** |
| **novel_737** | **2** | **5** | **3** | **3** | **1** | **3** |
| **novel_738** | **2** | **0** | **0** | **0** | **0** | **0** |
| **novel_740** | **1** | **0** | **1** | **3** | **2** | **2** |
| **novel_743** | **1** | **1** | **2** | **5** | **2** | **1** |
| **novel_745** | **0** | **0** | **1** | **0** | **0** | **0** |
| **novel_747** | **0** | **2** | **0** | **0** | **0** | **1** |
| **novel_750** | **5** | **5** | **0** | **1** | **0** | **1** |
| **novel_754** | **329** | **397** | **475** | **345** | **417** | **281** |
| **novel_759** | **1** | **4** | **0** | **0** | **0** | **0** |
| **novel_764** | **4** | **1** | **2** | **0** | **0** | **2** |
| **novel_768** | **17** | **8** | **10** | **20** | **18** | **20** |
| **novel_771** | **4** | **5** | **7** | **2** | **7** | **4** |
| **novel_772** | **0** | **2** | **0** | **1** | **0** | **1** |
| **novel_773** | **24** | **21** | **35** | **20** | **21** | **13** |
| **novel_777** | **0** | **2** | **3** | **0** | **2** | **1** |
| **novel_778** | **4** | **5** | **3** | **6** | **5** | **1** |
| **novel_78** | **746** | **1030** | **1112** | **1135** | **1020** | **806** |
| **novel_784** | **3** | **5** | **2** | **1** | **2** | **1** |
| **novel_785** | **4** | **0** | **1** | **3** | **0** | **1** |
| **novel_786** | **2** | **3** | **2** | **1** | **2** | **3** |
| **novel_787** | **0** | **1** | **1** | **2** | **0** | **2** |
| **novel_790** | **3** | **1** | **1** | **0** | **2** | **2** |
| **novel_791** | **0** | **1** | **2** | **2** | **0** | **1** |
| **novel_793** | **1** | **3** | **2** | **3** | **0** | **2** |
| **novel_794** | **0** | **0** | **0** | **0** | **1** | **0** |
| **novel_798** | **0** | **0** | **2** | **1** | **0** | **1** |
| **novel_800** | **8** | **4** | **2** | **6** | **1** | **2** |
| **novel_801** | **35** | **44** | **42** | **46** | **38** | **26** |
| **novel_803** | **0** | **0** | **1** | **0** | **0** | **0** |
| **novel_806** | **1** | **1** | **1** | **1** | **1** | **0** |
| **novel_808** | **0** | **2** | **1** | **0** | **3** | **1** |
| **novel_814** | **1** | **1** | **1** | **0** | **0** | **2** |
| **novel_816** | **0** | **1** | **0** | **0** | **0** | **0** |
| **novel_817** | **10** | **4** | **6** | **8** | **8** | **5** |
| **novel_819** | **4** | **7** | **0** | **2** | **4** | **1** |
| **novel_820** | **0** | **0** | **0** | **1** | **0** | **0** |
| **novel_822** | **9** | **14** | **17** | **11** | **10** | **5** |
| **novel_828** | **1** | **5** | **9** | **4** | **4** | **2** |
| **novel_831** | **0** | **2** | **0** | **1** | **0** | **0** |
| **novel_832** | **7** | **8** | **10** | **14** | **7** | **9** |
| **novel_833** | **1** | **1** | **0** | **2** | **3** | **2** |
| **novel_842** | **1** | **0** | **2** | **1** | **0** | **2** |
| **novel_845** | **0** | **0** | **1** | **0** | **0** | **0** |
| **novel_849** | **1** | **1** | **0** | **1** | **1** | **0** |
| **novel_854** | **6** | **3** | **7** | **3** | **4** | **4** |
| **novel_855** | **0** | **0** | **1** | **1** | **0** | **1** |
| **novel_858** | **1** | **4** | **0** | **1** | **2** | **1** |
| **novel_861** | **1** | **4** | **0** | **3** | **3** | **1** |
| **novel_862** | **0** | **0** | **0** | **0** | **0** | **1** |
| **novel_863** | **1** | **0** | **0** | **1** | **0** | **2** |
| **novel_868** | **7** | **17** | **6** | **6** | **10** | **2** |
| **novel_870** | **2** | **1** | **1** | **1** | **3** | **2** |
| **novel_872** | **0** | **1** | **2** | **0** | **0** | **2** |
| **novel_875** | **1** | **0** | **0** | **3** | **2** | **1** |
| **novel_876** | **0** | **0** | **0** | **1** | **1** | **0** |
| **novel_878** | **11** | **11** | **8** | **10** | **5** | **4** |
| **novel_880** | **3** | **1** | **1** | **2** | **1** | **0** |
| **novel_883** | **0** | **1** | **1** | **1** | **1** | **1** |
| **novel_886** | **1** | **0** | **0** | **0** | **0** | **1** |
| **novel_890** | **0** | **0** | **0** | **1** | **0** | **0** |
| **novel_897** | **1** | **1** | **1** | **0** | **0** | **1** |
| **novel_899** | **0** | **0** | **1** | **1** | **0** | **0** |
| **novel_901** | **5** | **8** | **10** | **6** | **10** | **9** |
| **novel_906** | **1** | **1** | **1** | **2** | **1** | **1** |
| **novel_911** | **22** | **21** | **21** | **11** | **20** | **7** |
| **novel_914** | **14** | **19** | **13** | **18** | **9** | **12** |
| **novel_920** | **0** | **2** | **0** | **1** | **1** | **3** |
| **novel_923** | **0** | **0** | **3** | **0** | **1** | **0** |
| **novel_925** | **6** | **5** | **4** | **7** | **6** | **9** |
| **novel_926** | **11** | **6** | **2** | **5** | **6** | **4** |
| **novel_927** | **1** | **6** | **6** | **5** | **3** | **0** |
| **novel_929** | **1** | **0** | **0** | **0** | **0** | **1** |
| **novel_932** | **1** | **0** | **0** | **0** | **2** | **0** |
| **novel_933** | **6** | **4** | **7** | **4** | **6** | **7** |
| **novel_935** | **124** | **94** | **87** | **98** | **118** | **87** |
| **novel_936** | **0** | **0** | **0** | **0** | **2** | **0** |
| **novel_937** | **4** | **0** | **0** | **2** | **0** | **0** |
| **novel_939** | **18** | **11** | **12** | **12** | **35** | **16** |
| **novel_940** | **8** | **18** | **14** | **9** | **8** | **6** |
| **novel_941** | **10** | **9** | **11** | **5** | **5** | **4** |
| **novel_943** | **0** | **1** | **0** | **1** | **1** | **0** |
| **novel_944** | **4** | **5** | **2** | **2** | **0** | **0** |
| **novel_945** | **1** | **4** | **3** | **4** | **6** | **2** |
| **novel_953** | **13** | **13** | **6** | **7** | **12** | **8** |
| **novel_954** | **2** | **1** | **1** | **0** | **1** | **0** |
| **novel_956** | **1** | **1** | **7** | **2** | **2** | **2** |
| **novel_957** | **9** | **7** | **11** | **7** | **12** | **7** |
| **novel_959** | **4** | **2** | **4** | **5** | **5** | **0** |
| **novel_962** | **0** | **0** | **0** | **3** | **2** | **0** |
| **novel_964** | **1** | **0** | **4** | **1** | **1** | **0** |
| **novel_965** | **1** | **1** | **2** | **1** | **0** | **1** |
| **novel_967** | **0** | **0** | **0** | **2** | **2** | **0** |
| **novel_969** | **3** | **8** | **1** | **3** | **2** | **5** |
| **novel_971** | **6** | **7** | **9** | **10** | **11** | **6** |
| **novel_974** | **1** | **0** | **0** | **1** | **3** | **0** |
| **novel_976** | **3** | **3** | **3** | **3** | **2** | **2** |
| **novel_978** | **0** | **1** | **1** | **2** | **2** | **0** |
| **novel_979** | **5** | **5** | **3** | **5** | **2** | **5** |
| **novel_980** | **7** | **4** | **10** | **8** | **17** | **7** |
| **novel_982** | **6** | **21** | **18** | **16** | **14** | **6** |
| **novel_983** | **6** | **4** | **6** | **3** | **6** | **5** |
| **novel_985** | **2** | **0** | **1** | **3** | **0** | **0** |
| **novel_998** | **3** | **1** | **1** | **0** | **1** | **0** |
