# Supplementary material for: PCV2 Regulates Cellular Inflammatory Responses through Dysregulating Cellular miRNA-mRNA Networks
Source: Viruses. 2019 Nov 13;11(11):1055. doi: 10.3390/v11111055 (PMC6893612; doi:10.3390/v11111055)
Supplement: Supplementary file 1 [file viruses-11-01055-s001.zip › Supplementary Files/Supplementary Table S8.docx]

**Supplementary Table S8.**

**Differentially expressed miRNAs between the PCV2-infected and mock-infected PK-15 cells.**

| **sRNA** | **Virus_read**  **count** | **Cell_read**  **count** | **log2Fold**  **Change** | **change** | **padj** |
| --- | --- | --- | --- | --- | --- |
| ssc-miR-450b-5p | 4185.861178 | 5639.971786 | -0.42749 | 0.743554296 | 1.74E-11 |
| ssc-miR-148b-3p | 34732.51438 | 46565.51069 | -0.41687 | 0.74904796 | 3.33E-05 |
| ssc-miR-184 | 2746.199488 | 3657.664958 | -0.40919 | 0.753046052 | 2.45E-06 |
| ssc-miR-26a | 18507.09377 | 24026.7493 | -0.37284 | 0.772260775 | 6.14E-06 |
| ssc-miR-374a-3p | 3052.848607 | 3932.700149 | -0.3629 | 0.777599933 | 2.59E-07 |
| ssc-miR-30a-5p | 469143.6133 | 598521.1343 | -0.34859 | 0.785351276 | 2.45E-06 |
| ssc-miR-30c-5p | 20643.74889 | 26213.74929 | -0.33963 | 0.790243955 | 0.0010824 |
| ssc-miR-10b | 102161.4035 | 129076.1122 | -0.334 | 0.793333843 | 6.64E-05 |
| ssc-miR-30a-3p | 9096.089851 | 11456.7045 | -0.32749 | 0.796921762 | 0.0026295 |
| ssc-miR-450a | 2234.914839 | 2802.150586 | -0.3241 | 0.798796546 | 1.32E-07 |
| novel_7 | 32100.70608 | 40263.40648 | -0.32397 | 0.798868528 | 3.38E-05 |
| ssc-miR-99a | 23048.11954 | 28662.42107 | -0.31064 | 0.806284001 | 0.0010598 |
| ssc-miR-489 | 209.2466767 | 259.0224981 | -0.3018 | 0.811239609 | 0.029824 |
| ssc-miR-450c-5p | 3229.741872 | 3984.979601 | -0.30019 | 0.812145431 | 0.00021264 |
| ssc-miR-128 | 8909.701173 | 10860.48428 | -0.28362 | 0.821527059 | 3.38E-05 |
| ssc-miR-10a-5p | 268024.1148 | 325990.1902 | -0.27995 | 0.823619561 | 0.00057215 |
| ssc-miR-30e-3p | 2283.301752 | 2764.404149 | -0.27052 | 0.829020683 | 0.022328 |
| ssc-miR-374b-5p | 3466.516757 | 4110.425343 | -0.24475 | 0.843962031 | 2.45E-06 |
| ssc-miR-499-5p | 751.6932321 | 893.4636824 | -0.24463 | 0.844032233 | 0.035684 |
| ssc-miR-27b-3p | 44955.25865 | 53242.75909 | -0.24117 | 0.846058895 | 0.014141 |
| ssc-let-7e | 5954.930151 | 6994.181458 | -0.23007 | 0.852593523 | 0.0075927 |
| ssc-miR-340 | 14030.55288 | 16355.89539 | -0.21891 | 0.859214354 | 0.019659 |
| ssc-let-7f | 151327.6029 | 176061.6312 | -0.21599 | 0.860955156 | 0.022226 |
| ssc-miR-30c-3p | 3673.762621 | 4250.392556 | -0.20906 | 0.865100711 | 0.0018742 |
| ssc-miR-30d | 107387.5685 | 123758.0241 | -0.20334 | 0.868537473 | 0.0047404 |
| ssc-miR-769-5p | 5053.715871 | 5659.837762 | -0.16215 | 0.893692238 | 0.020441 |
| ssc-miR-361-3p | 2509.866461 | 2807.142902 | -0.16145 | 0.894125966 | 0.017185 |
| ssc-miR-429 | 6943.364232 | 7721.146341 | -0.15268 | 0.899577823 | 0.020441 |
| ssc-miR-21 | 2587658.538 | 2302818.737 | 0.16787 | 1.12339867 | 2.78E-05 |
| ssc-miR-222 | 21824.82575 | 18847.59722 | 0.20937 | 1.156183188 | 0.021203 |
| ssc-miR-6782-3p | 780.4823555 | 647.6031877 | 0.2651 | 1.201719343 | 0.0018455 |
| ssc-miR-1307 | 5349.328582 | 4430.972933 | 0.26775 | 1.203928738 | 0.01555 |
| ssc-miR-24-3p | 17810.24109 | 14705.05264 | 0.27261 | 1.207991246 | 0.010191 |
| ssc-miR-193a-5p | 813.0769156 | 669.9032891 | 0.27666 | 1.211387139 | 0.0014338 |
| ssc-miR-320 | 5423.909983 | 4459.248278 | 0.27739 | 1.212000253 | 0.020441 |
| ssc-miR-221-3p | 35940.1788 | 29305.27266 | 0.28846 | 1.221335871 | 0.022328 |
| ssc-miR-378 | 26893.73161 | 21246.70875 | 0.32914 | 1.256264284 | 0.042567 |
| ssc-miR-29b | 460.4629468 | 363.7149892 | 0.33052 | 1.25746653 | 0.022328 |
| ssc-miR-671-5p | 278.9700172 | 218.4049119 | 0.34208 | 1.26758281 | 0.021112 |
| ssc-miR-378b-3p | 2490.751766 | 1904.661787 | 0.37965 | 1.301026186 | 0.0018742 |
| ssc-miR-130b | 164.63712 | 118.6608293 | 0.45553 | 1.371286479 | 0.011203 |
| ssc-miR-210 | 2346.623408 | 1692.21447 | 0.45937 | 1.374941274 | 0.00074673 |
| ssc-miR-146a-5p | 2060.913843 | 1466.327627 | 0.47719 | 1.392029707 | 0.00073074 |
| ssc-miR-148a-5p | 127.0073591 | 84.92387244 | 0.54762 | 1.461672399 | 0.01555 |
